# Establishing Predictors of Acute Sarcopenia: A Proof-Of-Concept Study Utilising Network Analysis

**DOI:** 10.14336/AD.2024.0167

**Published:** 2025-06-11

**Authors:** Carly Welch, Laura Bravo, Georgios Gkoutos, Carolyn Greig, Danielle Lewis, Janet Lord, Zeinab Majid, Tahir Masud, Kirsty McGee, Hannah Moorey, Thomas Pinkney, Benjamin Stanley, Thomas Jackson

**Affiliations:** ^1^Medical Research Council (MRC) - Versus Arthritis Centre for Musculoskeletal Ageing Research, University of Birmingham and University of Nottingham, UK.; ^2^Institute of Inflammation and Ageing, College of Medical and Dental Sciences, University of Birmingham, Birmingham, B152TT, UK.; ^3^University Hospitals Birmingham NHS Foundation Trust, Birmingham, B152GW, UK.; ^4^Department of Twin Research & Genetic Epidemiology, King’s College London, St Thomas’ Campus, London, SE1 7EH, UK.; ^5^Department of Ageing and Health, Guy’s and St Thomas’ NHS Foundation Trust, St Thomas’ Hospital, SE1 7EH, UK.; ^6^Institute of Cancer and Genomic Sciences, College of Medical and Dental Sciences, University of Birmingham, Birmingham, B152TT, UK.; ^7^School of Sport, Exercise, and Rehabilitation Sciences, University of Birmingham, Birmingham, B152TT, UK.; ^8^Birmingham Biomedical Research Centre, University of Birmingham and University Hospitals Birmingham NHS Foundation Trust, Birmingham, UK.; ^9^Nottingham University Hospitals NHS Trust, Nottingham, UK.; ^10^University of Nottingham, Nottingham, UK.; ^11^Institute of Applied Health Research, College of Medical and Dental Sciences, Birmingham Clinical Trials Unit, Public Health Building, University of Birmingham, Edgbaston, Birmingham, B15 2TT, UK.

**Keywords:** COPD, Steroids, Ultrasound, Delirium, IL-7

## Abstract

Dynamic changes in sarcopenia status following stressor events are defined as acute sarcopenia; it is currently unknown how to stratify risk. Prospective observational study involving elective colorectal surgery, emergency abdominal surgery, and medical patients with infections aged ≥70 years-old. Handgrip strength, muscle quantity (ultrasound Bilateral Anterior Thigh Thickness, BATT, and Bioelectrical Impedance Analysis), and muscle quality (rectus femoris echogenicity) were measured preoperatively in the elective group, and within 48hours, 7days after, and 13weeks after admission/surgery. Serum/plasma samples were collected preoperatively (elective group) and within 48hours of admission/surgery (all groups). LASSO models adjusting for baseline sarcopenia status were performed. Seventy-nine participants were included (mean age 79.1, 39.2% female). Chronic Obstructive Pulmonary Disease (COPD) (48hours β 0.67, CI 0.59-0.75), and prescription of steroids during admission (48hours β 1.11, CI 0.98-1.24) were positively associated with sarcopenia at 7days. Delirium was negatively associated with change in BATT to 7days (7days β -0.47, CI -0.5- -0.44). COPD (Preoperative β 0.35, CI 0.12-0.58) and delirium (48hours β 0.13, CI 0.06-0.2) were positively associated with change in echogenicity to 7days in analysis including systemic biomarkers. Participants with sarcopenia at baseline had higher IL-7 concentrations during acute phase of illness (median 8.78pg/mL vs 6.52pg/mL; p=0.014). IL-1b within 48hours of admission/surgery was positively associated with sarcopenia status at 7days (β 0.24, CI 0.06-0.42). Patients most at risk of acute sarcopenia or reductions in muscle quantity and quality included those prescribed steroids, with COPD or delirium, or with heightened systemic inflammation.

## INTRODUCTION

Acute sarcopenia (acute muscle insufficiency [1]), is recognised as an important emergent diagnosis, particularly affecting hospitalised older adults [2, 3]. It is defined by the development of incident sarcopenia (i.e. low muscle strength with low muscle quantity or quality) within six months, normally following a stressor event [4]. Recent studies have characterised changes in muscle quantity, quality, and function in hospitalised populations [5-7], with further studies ongoing or proposed. However, few studies have evaluated the relationship of systemic biomarkers with the development of sarcopenia, or assessed how predisposing or precipitating factors may cluster to increase risk and enable treatment stratification. In chronic sarcopenia, biomarkers associated with sarcopenia prevalence include myostatin [8], inflammatory cytokines [8], and Growth Hormone (GH)/Insulin-like Growth Factor 1 (IGF-1). Delirium is an acute neuropsychiatric disorder that occurs commonly secondary to acute illness in older adults, and which is associated with systemic inflammation [9]. However, patients with delirium have been frequently excluded from trials of interventions to combat negative changes in muscle quantity and physical function in hospitalised older people [10]. This study aimed to enhance understanding of how time-dependent biomarkers and patient-related factors may relate to acute sarcopenia risk.

## MATERIALS AND METHODS

### Study setting and design

This was a single centre study conducted at the Queen Elizabeth Hospital Birmingham, UK. The original protocol for the study was published previously [11] and the study was prospectively registered (NCT03858192). Participants aged 70 years and older were recruited to three groups: elective surgery (participants planned to undergo a major colorectal surgery procedure), emergency surgery (participants who had undergone an emergency abdominal procedure), and medical (admitted with acute bacterial infections or Coronavirus 2019, COVID-19). Participants provided informed consent or consultee declaration was obtained if they were considered to lack capacity to consent for themselves. Elective surgery participants were recruited from preoperative assessment clinic, and emergency surgery and medical participants were recruited from surgical and medical wards respectively. Exclusion criteria included inability to understand written or verbal English, and life expectancy less than 30 days.

### Research procedures

Baseline assessments were performed in preoperative assessment clinic in elective surgery participants, within 48 hours of surgery in emergency surgery participants, and within 48 hours of admission in medical participants. All assessments were performed by a clinician with training and expertise in geriatric medicine. Further assessments were performed within 48 hours post-operatively in the elective group, 7 (+/-2) days post-admission/post-operatively, and 13 (+/-1) weeks post-admission/post-operatively. Assessments performed at each timepoint included handgrip strength, ultrasound quadriceps, and Bioelectrical Impedance Analysis (BIA). Ultrasound (Venue 50, GE Healthcare) of the quadriceps was performed on both sides at the midpoint between the greater trochanter at the hip and the lateral joint line of the knee. The thickness of the rectus femoris (RF) and vastus intermedius (VI) muscles was measured on serial images not including the fascia in the transverse plane. The average of each thickness measurement was used for analysis. Bilateral Anterior Thigh Thickness (BATT) was calculated as the total thickness of the right RF + right VI + left RF + left VI [11, 12]. A single image was taken on each side in the longitudinal plane and RF echogenicity was calculated using grey scale analysis using Image J software [11, 12]. BIA was performed using a Bodystat Quadscan 4000 device. Skeletal Muscle Mass (SMM) was estimated according to the Sergi [13] and Janssen [14] equations. Phase angle was extracted directly from the device. Short Physical Performance Battery (SPPB) [15] was measured at 13 weeks in the emergency surgery group, at baseline and at 13 weeks in the elective surgery group, and at all timepoints in the medical group. Gait speed alone was measured at 7 days in the surgical groups.

### Sarcopenia definition

Sarcopenia was defined as low handgrip strength (<16kg in females, <27kg in males), with low BATT (<3.85cm in females, <5.44cm in males) and/or low SMM (<15kg in females, <20kg in males). The presence of sarcopenia was defined at each timepoint.

### Other clinical information

Demographic data, smoking status, binary coded individual long-term conditions, and binary coded treatments given were collected from the participant and/or patient records. Nutrition was assessed using the Mini-Nutritional Assessment (MNA) at baseline and at 13 weeks. In-hospital step count was recorded using Fitbit Inspire devices (Fitbit, Inc., Google LLC, USA). Delirium was recorded as assessed by the investigating geriatrician at each timepoint, according to the Diagnostic and Statistical Model of Diseases 5 (DSM-5) [16], or if a diagnosis of delirium was made by the patient’s own clinicians at any time during admission. Participant reported physical function was recorded through the Patient-Reported Outcome Measures Information System (PROMIS®) physical function 10 questionnaire [17]. Other variables recorded included length of hospital stay, and hospital readmission with total time spent in hospital.

### Measurement of systemic biomarkers

Selected biomarkers performed as part of routine clinical care were recorded at each timepoint where available (haemoglobin, creatinine, estimated Glomerular Filtration Rate - eGFR, C-Reactive Protein - CRP, albumin, white cell count, neutrophil count, and lymphocyte count). Additional blood samples were obtained within 48 hours of admission or surgery in all groups, and preoperatively in the elective surgery group. Plasma cortisol concentration was measured using Human Cortisol ELISA Kit (E-EL-0157, Elabscience), plasma Dehydroepiandrosterone sulfate (DHEA-s) concentration was measured using Human DHEA-s ELISA Kit (EH2946, FineTest, Wuhan Fine Biotech Co., Ltd.), serum High sensitivity CRP (hsCRP) concentration was measured using Human hsCRP ELISA Kit (HK369, HycultBiotech), serum Growth Hormone (GH) concentration was measured using Human Growth Hormone sandwich ELISA kit (KE00167, Proteintech), serum Insulin-like Growth Factor 1 (IGF-1) was measured using Human IGF-1 ELISA Kit (ELH-IGF1, RayBiotech), serum myostatin concentration was measured using Human Myostatin ELISA Kit (DL-MSTN-Hu, Dldevelop), and plasma total 25-hydroxy Vitamin D was measured using Total 25-OH Vitamin D ELISA Kit (80987, Crystal Chem). Serum concentrations of CCL2/JE/MCP-1, CXCL1/GRO alpha/KC/CINC-1, Flt-2 Ligand/FLT3L, IL-1 alpha/IL-1F1, IL-4, IL-7, IL-10, TNF-alpha, CCL3/MIP-1 alpha, CXCL10/IP-10/CRG-2, IFN-gamma, IL-1 beta/IL-1F2, IL-6, IL-8/CXCL8, IL-15, and VEGF were measured using Human XL Cytokine Premixed Luminex Performance Assay Kit (1621325, R&D systems, Bio-techne). Resistin and leptin were measured using Human Obesity Premixed Magnetic Luminex Performance Assay Kit (P205396, R&D systems, Bio-techne). Full methodology is included in the online supplement.

### Statistical analysis

Data description: A full list of variables initially included within the analysis is available in the online supplement (Supplementary Tables 1-2). This study represents a substudy of the original study; the study was not initially powered for analysis of systemic biomarkers. The original sample size calculation was derived in order to detect clinically significant change in muscle quantity and physical function variables within groups. Due to the COVID-19 pandemic, the sample size calculation was revised in order to enable detection of differences across groups. Baseline characteristics are displayed in text and tables, separated by patient group. Significance of differences were analysed using one-way Analysis of Variance (ANOVA), Chi-squared, Kruskal-Wallis, and Wilcoxon Rank Sum tests. Mean and median values of systemic biomarkers are displayed in table format (Supplementary Table 3). Statistical significance of differences between participants with and without sarcopenia at baseline and at 7 days were analysed using unpaired t-tests and Wilcoxon Rank Sum tests as applicable. A heatmap showing all missing values is shown in Supplementary Figure 1.

Modelling: Advanced data modelling was performed by a bioinformatician (LB) who was independent from clinical and laboratory data collection. Least Absolute Shrinkage and Selection Operator (LASSO) modelling is a penalised regression model able to shrink covariate coefficients towards zero, allowing for the generation of sparse models and concurrently performing feature selection [18]. In this study, LASSO has been applied for both classification and regression to consider prediction of categorical and numerical variables respectively. Firstly, LASSO was applied on data from each timepoint with “SarcAny” as the outcome for classification analysis adjusting for baseline sarcopenia status. Then, information on "Echo", "BATT", and "SMMSergi" at each time point were predicted through regression and their changes from baseline to 7 days, as well as baseline to 13 weeks. In each of these models, data collected at previous timepoints were used to predict future outcomes and due to small sample size of systemic biomarker data, two different analyses were performed: 1) including all data, deleting all features with 30% or more of missing values and imputing those remaining with the median (numerical) or mode (categorical) or, 2) focusing on participants who had systemic biomarker data specifically and imputing any missing values with the median (numerical) or mode (categorical). In total, 64 different models were built, studying the four mentioned outcomes (“SarcAny”, "Echo", "BATT", and "SMMSergi") at each specific timepoint, using all different timepoint data. Moreover, each of those 64 models was bootstrapped from 20 to 70 times, depending on their sample sizes. The number of times features were "selected " in each of the models was counted, and those above the threshold (mean between maximum selected feature and third quartile) had their coefficients averaged and confidence intervals calculated [19]. More information on data sample sizes and coefficient selection are shown in Table 1 and Supplementary Tables 4-6. Networks were created through igraph [20] and Cytoscape [21] by combining all of the selected features and outcomes for each data point and using the averaged coefficients as weights. The full code is available at: https://github.com/InFlamUOB/Sarcopenia.

**Table 1:** Baseline characteristics for participants separated by patient cohort.

		Overall (N=79)	Elective surgery (N=24)	Emergency surgery (N=14)	Medical (N=41)	p value
**Age - mean (SD)**		79.1 (6.6)	76.4 (5.3)	75.2 (4.2)	82.1 (6.7)	<0.001^a^
**Gender - Females % (N)**		39.2 (31)	50.0 (12)	35.7 (5)	34.1 (14)	0.431^b^
**Ethnicity - % (N)**	White British	93.7 (74)	95.8 (23)	100 (14)	90.2 (37)	0.742^b^
	White Irish	2.5 (2)	0 (0)	0 (0)	4.9 (2)	
	Indian	2.5 (2)	4.2 (1)	0 (0)	2.4 (1)	
	Arab	1.3 (1)	0 (0)	0 (0)	2.4 (1)	
**Body Mass Index (kg/m^2^) - mean (SD)**		26.5 (6.5)	26.4 (4.3)	24.3 (4.3)	27.4 (8.0)	0.303^a^
**Nutritional status - % (N)**	Normal	41.8 (33)	75.0 (18)	35.7 (0)	24.4 (10)	0.001^b^
	At risk	50.6 (40)	25.0 (6)	64.3 (9)	61.0 (25)	
	Malnourished	7.6 (6)	0 (0)	0 (0)	14.6 (6)	

a One-way ANOVA;

b Chi-squared test;

c Kruskal-Wallis test;

d Wilcoxon Rank Sum test

## RESULTS

Seventy-nine participants were recruited to the study and included within this analysis. Recruitment and drop-out rates are shown in Supplementary Figure 1. Full feasibility analysis including screening and recruitment rates has been published previously [22]. The mean age of participants was 79.1 (6.6) and 39.2% (31/79) were female. Baseline characteristics of participants are shown in Table 1. Blood samples were collected for research purposes for all elective participants (24/24), and within 48 hours of admission/surgery for 64.6% (51/79).

### Clinical features

In analysis without systemic biomarkers, clinical features that were shown to be positively associated with sarcopenia status at 7 days (adjusting for baseline sarcopenia status) were anxiety/depression (preoperative β 0.44, CI 0.17 - 0.72), asthma (48 hours β 0.77, CI 0.61 - 0.92), Chronic Obstructive Pulmonary Disease (COPD) across all timepoints (48 hours β 0.67, CI 0.59 - 0.75), Ischaemic Heart Disease (7 days β 0.7, CI 0.55 - 0.85), and prescription of steroids during admission (48 hours β 1.11, CI 0.98 - 1.24) (Table 1). White British ethnicity was negatively associated with sarcopenia status at 13 weeks (13 weeks β -0.24, CI -0.32 - -0.15). Clinical features included within sarcopenia diagnosis were negatively associated with sarcopenia status at each timepoint (BATT, SMMSergi, and handgrip strength). Similar patterns were demonstrated in analysis including only participants with systemic biomarkers available (Supplementary Table 4). Patient-reported physical function (PROMIS physical function) at preoperative assessment was negatively associated with sarcopenia status at 7 days.

The presence of delirium was negatively associated with change in BATT to 7 days (7 days β -0.47, CI -0.5 - -0.44), in analysis not including systemic biomarkers (Supplementary Table 5). COPD was positively associated with change in BATT to 7 days (7 days β 0.23, CI 0.21 - 0.25). Ischaemic Heart Disease (48 hours β -0.38, CI -0.47 - -0.29) and prescription of metformin were negatively associated with change in SMMSergi to 7 days (48 hours β -0.54, CI -0.67 - -0.41). Diabetes Mellitus was positively associated with change in SMMSergi to 7 days (48 hours β 0.48, CI 0.38 - 0.57). These associations were not replicated in analysis including systemic biomarkers, although COPD (Preoperative β 0.35, CI 0.12 - 0.58), delirium (48 hours β 0.13, CI 0.06 - 0.2), and metformin prescription (Preoperative β 0.21, CI 0.13 - 0.28) were positively associated with change in echogenicity to 7 days (Supplementary Table 6).

### Systemic biomarkers

Supplementary Table 3 shows mean/median concentrations of systemic biomarkers separated according to sarcopenia status at baseline and at 7 days. Preoperative biomarkers in the elective cohort are presented separately from biomarkers measured within 48 hours of admission/surgery. There were few statistically significant differences between participants with and without sarcopenia in this unadjusted analysis, although some differences appeared to be clinically significant (e.g. lower GH concentrations in participants with sarcopenia at all timepoints). Participants who met criteria for sarcopenia at baseline had significantly higher IL-7 concentration measured during the acute phase of illness (median 8.78pg/mL vs 6.52pg/mL; p=0.014).

**Table 2:** Beta coefficients derived from LASSO and Elastic Net models for outcomes at timepoints, without specific focus on participants with additional systemic biomarkers available.

	Timing	BATT (7 days)	SMMSergi (7 days)	Echogenicity (7 days)	Sarcopenia (7 days)	Sarcopenia (13 weeks)
**Age**	Preop			0.11 [0.06, 0.16] (18/36)		
	13 weeks				0.14 [0.02, 0.27] (9/22)	0.12 [0.02, 0.21] (5/23)
**Anxiety/Depression**	Preop		0.11 [0.04, 0.17] (9/36)		0.44 [0.17, 0.72] (11/18)	
	7 days		0.22 [0.17, 0.27] (40/79)			
**Asthma**	Preop			0.62 [0.44, 0.81] (24/36)		
	48 hours				0.77 [0.61, 0.92] (64/70)	
	7 days				0.75 [0.62, 0.88] (52/70)	
**BATT** **(48 hours)**	48 hours				-0.17 [-0.23, -0.1] (52/70)	
**BATT** **(7 days)**	7 days				-0.23 [-0.26, -0.2] (46/70)	
**BATT** **(13 weeks)**	13 weeks					-0.23 [-0.35, -0.1] (12/23)
**Cancer**	Preop			-0.22 [-0.29, -0.16] (25/36)		-0.41 [-0.53, -0.3] (11/20)
**COPD**	Preop			0.61 [0.51, 0.72] (25/36)	1.05 [0.86, 1.24] (16/18)	-0.47 [-0.7, -0.25] (12/20)
	48 hours	-0.32 [-0.34, -0.29] (77/79)	-0.29 [-0.31, -0.26] (68/79)		0.67 [0.59, 0.75] (66/70)	
	7 days	-0.36 [-0.38, -0.33] (78/79)	-0.31 [-0.34, -0.28] (65/79)		0.89 [0.71, 1.06] (63/70)	
	13 weeks	-0.67 [-0.7, -0.64] (78/79)	-0.56 [-0.6, -0.53] (79/79)		1.37 [1.09, 1.66] (22/22)	
**Creatinine (Preop)**	Preop	0.17 [0.15, 0.2] (36/36)	0.25 [0.19, 0.31] (22/36)			
**CRP (48 hours)**	48 hours	-0.04 [-0.05, -0.03] (34/79)				
**Delirium**	13 weeks				-1.15 [-1.95, -0.35] (8/22)	
**Digoxin**	13 weeks				-1.42 [-2.02, -0.82] (20/22)	
**Diabetes Mellitus**	48 hours	0.39 [0.36, 0.43] (79/79)	0.13 [0.1, 0.16] (49/79)			
	7 days	0.41 [0.38, 0.43] (79/79)	0.14 [0.12, 0.17] (53/79)			
	13 weeks	0.42 [0.39, 0.45] (77/79)	0.16 [0.13, 0.2] (65/79)		-0.72 [-1.02, -0.42] (13/22)	-0.64 [-0.78, -0.5] (13/23)
**eGFR** **(48 hours)**	48 hours	-0.05 [-0.06, -0.04] (53/79)	-0.12 [-0.14, -0.11] (64/79)			
**eGFR (7days)**	7 days	-0.16 [-0.17, -0.15] (79/79)	-0.21 [-0.23, -0.2] (74/79)			
**eGFR (Preop)**	Preop			0.39 [0.27, 0.5] (24/36)		
**White British ethnicity**	13weeks					-0.24 [-0.32, -0.15] (15/23)
**Hb (7 days)**	7 days	-0.05 [-0.07, -0.04] (35/79)				
**Handgrip strength (48 hours)**	48 hours	0.22 [0.21, 0.23] (79/79)	0.17 [0.16, 0.18] (75/79)		-0.91 [-1.01, -0.81] (70/70)	
**Handgrip strength** **(7 days)**	7 days	0.28 [0.27, 0.29] (79/79)	0.26 [0.25, 0.27] (75/79)		-1.25 [-1.42, -1.08] (70/70)	
**Handgrip strength (13 weeks)**	13 weeks				-0.63 [-0.86, -0.39] (21/22)	-0.57 [-0.74, -0.41] (17/23)
Ischaemic Heart Disease	48 hours	0.16 [0.14, 0.19] (62/79)				
	**7 days**	0.21 [0.18, 0.24] (62/79)			0.7 [0.55, 0.85] (40/70)	
	**13 weeks**	0.28 [0.25, 0.3] (73/79)				
**Length of stay**	Preop			-0.16 [-0.24, -0.08] (19/36)		
**Lymphocytes** **(Preop)**	Preop			-0.13 [-0.16, -0.09] (22/36)		
**Lymphocytes** **(7 days)**	7 days	-0.06 [-0.07, -0.04] (47/79)				
Metformin	Preop			1.11 [0.8, 1.42] (25/36)		
	**7 days**	0.25 [0.18, 0.31] (36/79)				
	**13 weeks**	0.2 [0.14, 0.26] (43/79)				
**Nutrition (13weeks): At Risk (vs malnourished)**	13 weeks				0.46 [0.32, 0.6] (21/22)	
**Phase Angle** **(13 weeks)**	13 weeks				-0.19 [-0.25, -0.14] (20/22)	-0.06 [-0.1, -0.02] (7/23)
**Phase Angle** **(48 hours)**	48 hours	0.06 [0.05, 0.06] (63/79)			-0.26 [-0.47, -0.06] (62/70)	
**Phase Angle** **(7 days)**	7 days	0.09 [0.08, 0.1] (72/79)			-0.58 [-0.73, -0.43] (47/70)	
**PROMIS Physical Function (Preop)**	Preop				-0.38 [-0.52, -0.25] (16/18)	
Sex (male)	Preop	0.1 [0.07, 0.13] (29/36)	0.08 [0.06, 0.11] (18/36)		0.2 [0.08, 0.33] (11/18)	-0.22 [-0.37, -0.07] (8/20)
	**48 hours**		0.15 [0.14, 0.17] (73/79)		0.27 [0.21, 0.34] (64/70)	
	**7 days**		0.12 [0.11, 0.14] (70/79)		0.56 [0.47, 0.65] (61/70)	
	**13 weeks**	0.15 [0.14, 0.17] (77/79)	0.33 [0.3, 0.35] (79/79)		0.84 [0.42, 1.26] (8/22)	0.45 [0.17, 0.74] (11/23)
**SMMSergi** **(13 weeks)**	13 weeks				-0.23 [-0.27, -0.18] (14/22)	
**Ex-smoker (vs current)**	Preop					-0.22 [-0.34, -0.09] (5/20)
Non-smoker (vs current)	Preop					-0.35 [-0.57, -0.14] (8/20)
	**7 days**	-0.15 [-0.19, -0.11] (39/79)				
	**13 weeks**	-0.19 [-0.22, -0.17] (59/79)				
Steroids	Preop			-0.25 [-0.36, -0.14] (21/36)	0.43 [0.26, 0.59] (10/18)	
	**48 hours**				1.11 [0.98, 1.24] (64/70)	
	**7 days**				0.75 [0.61, 0.89] (58/70)	
	**13 weeks**				1.18 [0.84, 1.52] (19/22)	
Stroke	7 days	0.23 [0.16, 0.3] (46/79)				
	**13 weeks**	0.21 [0.12, 0.3] (33/79)				
**White Cell Count** **(Preop)**	Preop			0.35 [0.13, 0.57] (24/36)		
**Walking Speed** **(13 weeks)**	13 weeks				-0.2 [-0.37, -0.04] (6/22)	

Results are adjusted for baseline sarcopenia status. Square brackets denote confidence intervals for coefficients. Curved brackets denote the number of models that the association was encountered within, and the number of models that the association was tested within. The timing of the individual variables and outcomes tested are denoted in the first column and row respectively. Variables without timing specified in the first column are constants. The separate timing (second) column refers to the timing of other variables that the associated was tested against. Non-significant associations are not shown.

IL-1b measured within 48 hours of admission/surgery was positively associated with sarcopenia status at 7 days (β 0.24, CI 0.06 - 0.42), and resistin was negatively associated (β -0.12, CI -0.23 - -0.01). TNFα measured both preoperatively and within 48 hours of admission/surgery was negatively associated with change in echogenicity and positively associated with change in SMMSergi to 7 days. Serum creatinine was positively associated with change in SMMSergi to 7 days; eGFR was negatively associated with change in BATT to 7 days.

### Network analysis

**Figure 1.**
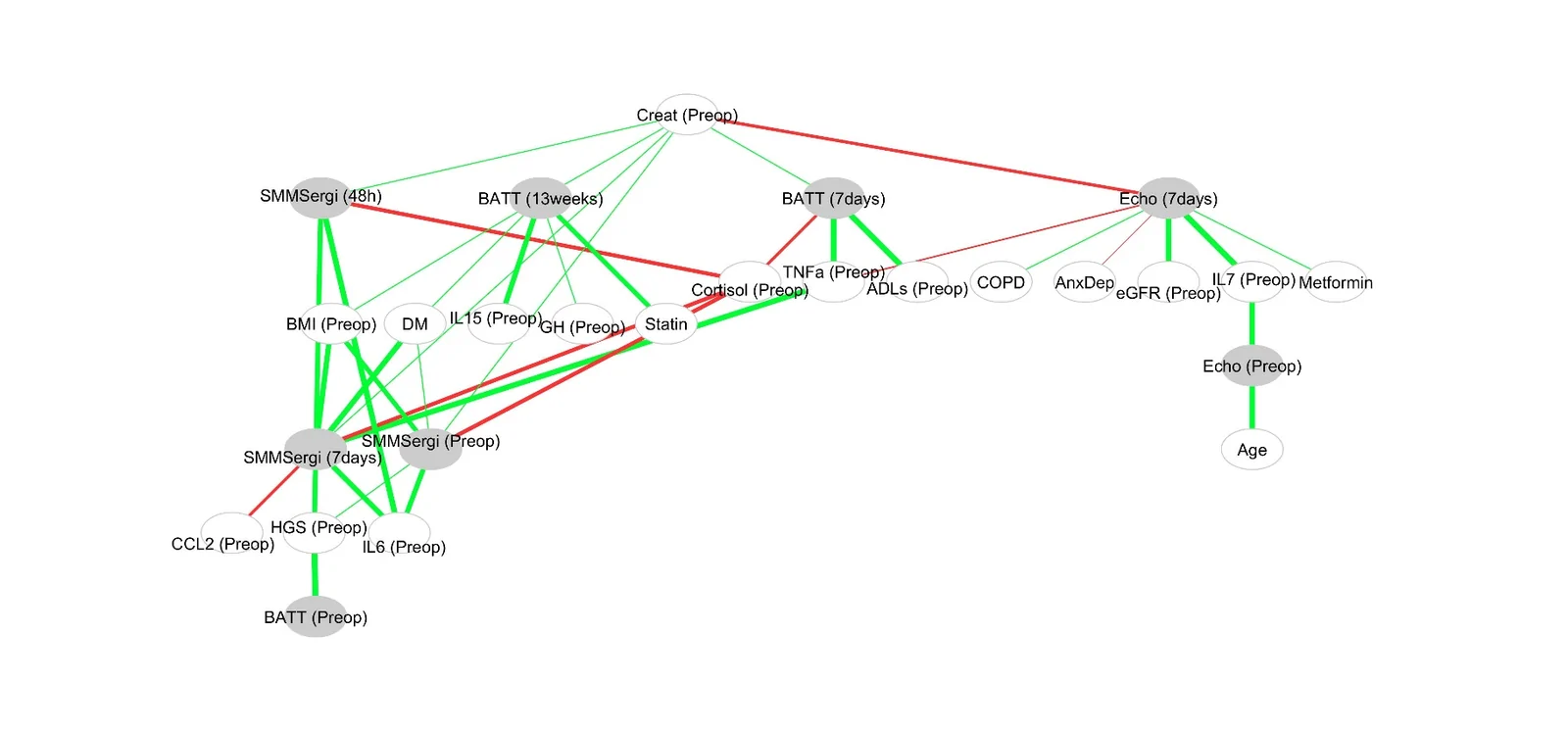
**Network derived from continuous variable outcomes including systemic biomarkers measured preoperatively**. Red lines show negative associations and green lines show positive associations. Echo=Echogenicity; BATT=Bilateral Anterior Thigh Thickness; SMMSergi=Skeletal Muscle Mass (Sergi equation); AnxDep=Anxiety/Depression; eGFR=estimated Glomerular Filtration Rate; COPD=Chronic Obstructive Pulmonary Disease; IL7=Interleukin 7; ADLs=Activities of Daily Living; TNFa=Tumour Necrosis Factor Alpha; IL15=Interleukin 15; GH=Growth Hormone; DM=Diabetes Mellitus; BMI=Body Mass Index; IL6=Interleukin 6; HGS=Handgrip Strength; CCL2=Chemokine (C-C motif) ligand 2.

**Figure 2.**
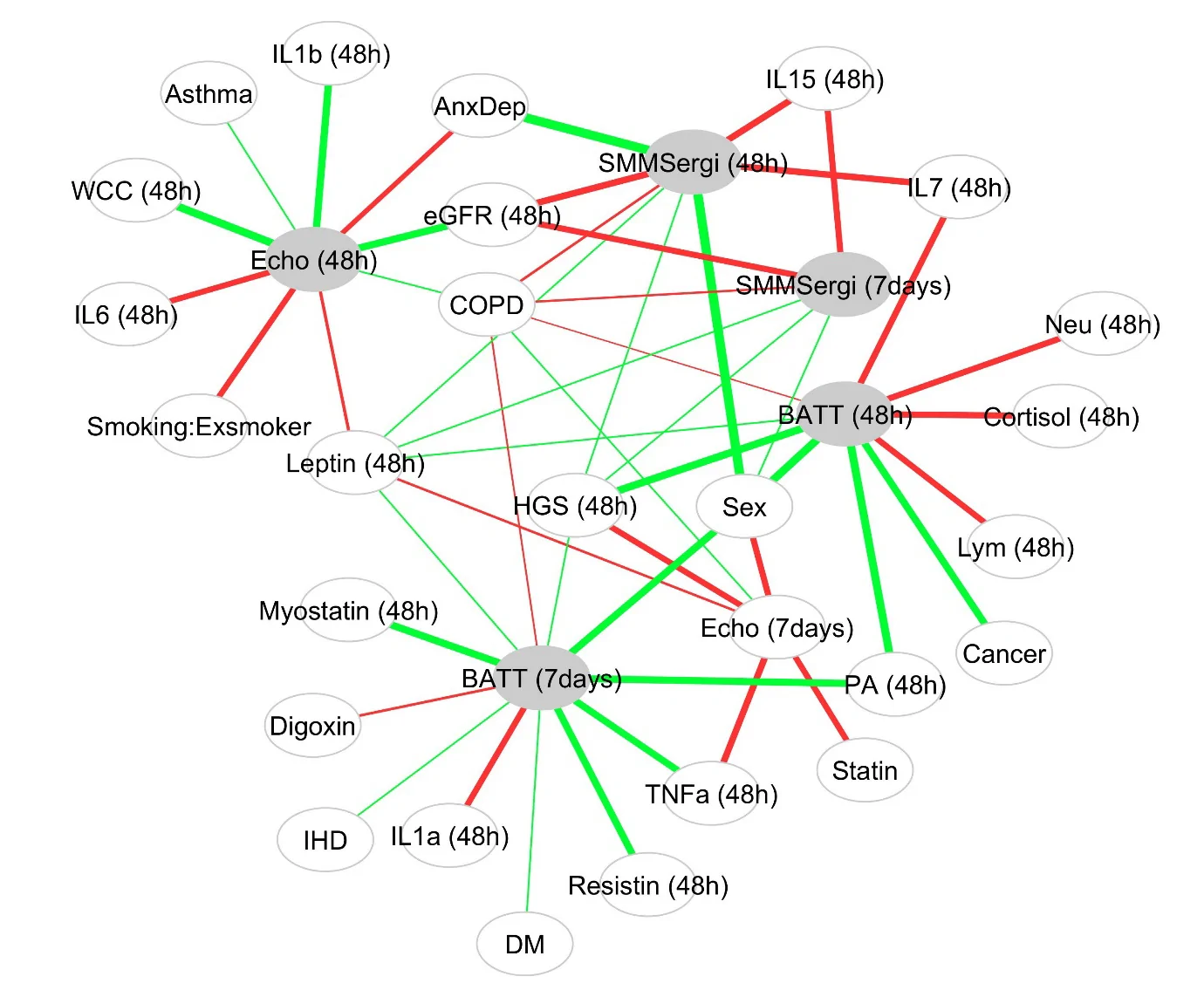
**Network derived from continuous variable outcomes including systemic biomarkers measured within 48 hours of surgery/admission**. Red lines show negative associations and green lines show positive associations. IL6=Interleukin 6; WCC=White Cell Count; IL1b=Interleukin 1 beta; AnxDep=Anxiety/Depression; Echo=Echogenicity; eGFR=estimated Glomerular Filtration Rate; COPD=Chronic Obstructive Pulmonary Disease; SMMSergi=Skeletal Muscle Mass (Sergi equation); IL15=Interleukin 15; IL7=Interleukin 7; BATT=Bilateral Anterior Thigh Thickness; Neu=Neutrophil count; HGS=Handgrip strength; Lym=Lymphocyte count; TNFa=Tumour Necrosis Factor Alpha; IL1a=Interleukin 1 Alpha; IHD=Ischaemic Heart Disease.

Figure 1 shows the network generated for outcomes including systemic biomarkers measured preoperatively. Preoperative IL7 was positively associated with echogenicity preoperatively and at 7 days. Preoperative TNFα was positively associated BATT and SMMSergi at 7 days. Variables associated with echogenicity appeared to cluster separately from variables associated with measures of muscle quantity. Figure 2 shows the network generated for outcomes including systemic biomarkers measured within 48 hours of surgery/admission. In this network, COPD showed consistent positive associations with echogenicity and negative associations with measures of muscle quantity.

## DISCUSSION

These results provide proof-of-concept towards the identification of clinical features and systemic biomarkers related to sarcopenia in hospitalised older patients, which will guide future research to enable clinical risk stratification and novel intervention strategies. COPD was consistently positively associated with sarcopenia status at 7 days in association with clinical features measured at all timepoints, in a high proportion of models. This association was demonstrated despite adjusting for baseline sarcopenia status, suggesting that this association may be distinct from any association with chronic sarcopenia. Conversely, COPD was positively associated with change in BATT, although it was positively associated with change in echogenicity i.e., increased muscle quantity but reduced muscle quality. Whilst echogenicity did not form part of the sarcopenia diagnosis used in this study, it is recognised that reduced muscle quality (i.e., elevated echogenicity) may be important to pathogenesis, and can be used in place of reduce muscle quantity in sarcopenia diagnosis [4]. Prescription of steroids at any point during admission was positively associated with sarcopenia at 7 days. This effect was also demonstrated consistently alongside clinical features measured at all timepoints and in high proportions of models. Steroid treatment has been shown to exacerbate loss of muscle quantity during bedrest in healthy adults [23] and upregulate pathways of muscle protein degradation in rodent models [24]. Patients with COPD are more likely to have been prescribed steroids acutely during admission as part of treatment for acute exacerbations, as well as to have received steroids previously, but there may also be separate innate common pathways within COPD aetiology.

Prescription of metformin was negatively associated with change in SMMSergi (in analysis without cytokines, in combination with clinical features at 48 hours) and positively associated with change in echogenicity (in analysis with cytokines, in combination with preoperative clinical features) to 7 days. This suggests that prescription of metformin may negatively impact on muscle quantity and quality. However, these effects were not consistent and there was no clear association with sarcopenia itself. Diabetes Mellitus was positively associated with change in SMMSergi and negatively associated with change in echogenicity, suggesting that the effects of metformin are distinct from any effect from Diabetes Mellitus. Metformin reduces inflammation and in rodent models has been shown to reduce fat infiltration within muscles following thermal injury [25]. On the other hand, evidence suggests that it may actually promote muscle protein breakdown and reduce muscle protein synthesis [26]. Studies are currently ongoing into the role of metformin in the treatment and prevention of chronic sarcopenia.

In our study there was a negative association with White British ethnicity and sarcopenia at 13 weeks. This suggests that patients who self-identify with other ethnic backgrounds may be at increased risk of poor recovery of muscle quantity and function following hospitalisation. The majority of participants recruited to this study were from a White British background, and we did not measure socioeconomic status as part of this study, which could account for these differences. However, this effect requires urgent further evaluation. Previous studies have demonstrated that older people who self-identify as belonging to a minority ethnic group have lower health-related quality of life compared to those who identify as White British [27].

Higher serum concentrations of IL-7 were measured during the acute phase of illness in participants who met criteria for sarcopenia at baseline. IL-7 is expressed and secreted by human skeletal muscle cells [28]. Whilst this process may be physiological, excessive secretion may lead to increased systemic inflammation and immune dysregulation. This suggests that chronic sarcopenia may be associated with dysregulated myokine secretion and immune adaptations. Sarcopenia has been consistently shown to be associated with increased risk of mortality and adverse outcomes [29], even when adjusting for factors such as comorbidities and functional status [30]. It is possible that these associations may relate to immune dysregulation directly precipitated by dysregulated muscle secretory processes in sarcopenia. Median GH concentrations were increased in the acute phase compared to preoperative levels in participants both with and without sarcopenia. However, concentrations remained consistently lower in participants with sarcopenia. This suggests that reduced baseline GH may lead to an ineffective surge with acute illness, and reduced promotion of muscle synthesis. GH is known to decline with age [31]. GH may increase with acute illness but with a state of peripheral GH resistance accompanied by low IGF-1 levels [32]. GH has been shown to promote muscle protein synthesis in healthy volunteers [33].

The presence of delirium was negatively associated with change in BATT and positively associated with change in echogenicity to 7 days (reduced muscle quantity and quality). These results are novel and merit further evaluation. Previous studies have shown that low baseline skeletal muscle mass is a risk factor for incident delirium [34], and that delirium is independently associated with risk of being sarcopenic upon admission to a geriatric unit [35]. However, we are not aware of previous studies that have assessed the association of changes in muscle quantity and quality with the presence of delirium. Delirium is considered to relate to processes of systemic inflammation and immune dysregulation [9]; these changes in turn may lead to increased risk of muscle protein breakdown. Additionally, delirium has been shown to be associated with reduced physical activity and prolonged bedrest during hospitalisation [36], which is known to be associated with increased risk of muscle wasting in older adults [37].

No systemic biomarkers were clearly and consistently associated with sarcopenia status at 7 days, or change in BATT, SMMSergi, or echogenicity. However, there was a positive association between IL-1b serum concentrations and sarcopenia status at 7 days. IL-1b is a pro-inflammatory cytokine secreted with acute inflammation. IL-1b has been shown to be expressed in myocytes in rodent models of sepsis and is considered to be a key mediator of muscle atrophy in this context [38]. Interestingly, serum and cerebrospinal fluid concentration levels of IL-1b are elevated in patients with delirium [39], which may explain the association demonstrated in this study between delirium and sarcopenia in the acute setting.

Interestingly, eGFR was shown to be negatively associated with change in BATT, and positively associated with change in echogenicity to 7 days. This would suggest that participants with better renal function had reduced muscle quantity and quality. However, creatinine was positively associated change in SMMSergi, and negatively associated with change in echogenicity at 7 days. Creatinine is a known biomarker of muscle quantity, as well as renal function, and the eGFR is derived from creatinine by the Modification of Diet in Renal Disease study equation [40]. It is counter-intuitive that improved renal function would be associated with reduced muscle quantity and quality, and it is more biologically plausible that this association relates to reduced serum creatinine levels with low muscle quantity. However, this suggests that the eGFR may be less reliable as a measure of renal function in patients with reduced skeletal muscle mass. The Cockcroft-Gault formula, which also considers the patient’s weight may be a more suitable alternative formula for estimation of renal function in older people at risk of sarcopenia [41].

### Strengths and limitations

This study presents results derived from clinical and laboratory-based research. All clinical assessments were completed by a clinician with training and experience in geriatric medicine. Statistical analysis was performed independently by a bioinformatician who was not involved in the collection of data for this study; robust methods were applied through the use of bootstrapping in model building. Demonstration of association of clinical features (BATT, SMMSergi, handgrip strength) used in the diagnosis of sarcopenia with sarcopenia status supports reliability of the models. However, it is important to note that the study was underpowered compared to the original planned sample size calculation. The exclusion of variables with greater than 30% missing values ensured robustness of the models but may have also led to exclusion of some variables that may have been of significance. Additionally, due to high numbers of participants where data were collected remotely at 13 weeks, many variables and outcomes were excluded from analysis at 13 weeks. Therefore, the results predominantly focus on biomarkers in relation to sarcopenia status at 7 days. The purpose of this analysis was to demonstrate associations towards proof-of-concept to guide future mechanistic, observational, and interventional studies. We, therefore, have not commented on the size or magnitude of significance of associations, which is the common approach for all network analyses. Further research should assess if results can be reproduced in a larger powered study incorporating greater consideration to magnitude of effect, as well as incorporation of results within future systemic reviews and meta-analyses. Simple unadjusted analyses were performed when comparing mean/median biomarker concentrations shown in Supplementary Table 3. These data are predominantly presented for descriptive purposes, but it is important to note that these differences do not account for differences between sex and patient groups. The low prevalence of participants from ethnic backgrounds other than White British is another limitation of this study.

### Recommendations for future research

Further mechanistic studies should aim to further assess inflammatory pathways involved in muscle atrophy in the acute setting. The role of IL-1b should be explored further and may potentially serve as a biomarker in risk stratification. Additionally, we recommend further studies consider enhanced measurement of other hallmarks of ageing across serial timepoints to assess how fundamental mechanisms relate to acute sarcopenia, delirium, and other clinical manifestations of impaired resilience following hospitalisation. To specifically enhance understanding of mechanisms driving acute sarcopenia collection of serial muscle biopsies to test upregulated myocellular pathways would be greatly beneficial. The results of this study did not clearly demonstrate potential interventions, but results may be used for comparison when designing and conducting trials including theoretical interventions related to the biomarkers measured in this study (e.g. GH injection, myostatin inhibitors). In considering how treatments are targeted to ensure greatest benefit, initially targeting treatment towards patients on treatment with steroid medication would be a pragmatic approach. This would include patients on treatment with prednisolone for exacerbations of COPD or asthma, as well as patients on treatment with dexamethasone for symptomatic COVID-19 infection. Patients with delirium are likely to be another group who would particularly benefit from targeted interventions, which will need to be carefully tailored to ensure effectiveness and feasibility in clinical practice.

We recommend that further larger powered studies should carefully consider recruitment methodology to consider how participants from under-represented groups such as those from minority ethnic backgrounds can be recruited to the study to ensure that results are representative across populations. Socioeconomic status should be prospectively recorded using surrogate markers such as the indices of multiple deprivation in England. Future research studies should carefully consider how to reduce and avoid missing data, although it is appreciated that this needs to be balanced against ensuring representativeness of the study population. The use of remote follow-up techniques can enable collection of valuable patient-reported outcome data even where in person review is not possible. Early involvement of bioinformaticians in development of the study protocol can ensure robust consideration of how missingness will be managed in planned statistical analysis. Where possible, longer follow-up beyond three months would provide further comprehensive data on the long-term progression of sarcopenia and recovery patterns.

### Conclusion

Acute sarcopenia is a complex phenomenon, and it is unlikely that a single biomarker would be sensitive or specific enough to identify or predict the onset of acute sarcopenia alone. No systemic biomarkers were consistently associated with both sarcopenia status at 7 days and changes in muscle quantity and quality at 7 days post-admission/surgery, although IL-1b was positively associated with sarcopenia status. Patients that may be considered most at risk include patients with heightened systemic inflammation, who are prescribed steroid medications, or diagnosed with delirium. Further mechanistic studies are warranted to elucidate underlying pathways to guide therapeutic interventions. At the same time, interventional studies should not be delayed and pragmatic studies of interventions with biological plausibility are encouraged.

## Supplementary Materials

The Supplementary data can be found online at: www.aginganddisease.org/EN/10.14336/AD.2024.0167.

## References

[1] Cruz-Jentoft A (2016). Sarcopenia, the last organ insufficiency. European Geriatric Medicine, 7:195-196.

[2] Welch C. 2021. Acute Sarcopenia: Definition and Actual Issues. In Sarcopenia: Research and Clinical Implications. Veronese N., Beaudart C., and Sabico S., editors. Cham: Springer International Publishing. 133-143.

[3] Welch C, Hassan-Smith ZK, Greig CA, Lord JM, Jackson TA (2018). Acute Sarcopenia Secondary to Hospitalisation - An Emerging Condition Affecting Older Adults. Aging and disease, 9:151-164. 10.14336/AD.2017.0315 PMC5772853 29392090

[4] Cruz-Jentoft AJ, Bahat G, Bauer J, Boirie Y, Bruyère O, Cederholm T, et al. (2019). Sarcopenia: revised European consensus on definition and diagnosis. Age Ageing, 48:16-31. 10.1093/ageing/afy169 PMC6322506 30312372

[5] Hartley P, Romero-Ortuno R, Wellwood I, Deaton C (2020). Changes in muscle strength and physical function in older patients during and after hospitalisation: a prospective repeated-measures cohort study. Age and Ageing, 50:153-160. 10.1093/ageing/afaa103 32902637

[6] Van Ancum JM, Scheerman K, Jonkman NH, Smeenk HE, Kruizinga RC, Meskers CGM, et al. (2017). Change in muscle strength and muscle mass in older hospitalized patients: A systematic review and meta-analysis. Exp Gerontol, 92:34-41. 10.1016/j.exger.2017.03.006 28286250

[7] Van Ancum JM, Scheerman K, Pierik VD, Numans ST, Verlaan S, Smeenk HE, et al. (2017). Muscle Strength and Muscle Mass in Older Patients during Hospitalization: The EMPOWER Study. Gerontology, 63:507-514. 10.1159/000478777 PMC5804856 28817825

[8] Patel HP, Al-Shanti N, Davies LC, Barton SJ, Grounds MD, Tellam RL, et al. (2014). Lean Mass, Muscle Strength and Gene Expression in Community Dwelling Older Men: Findings from the Hertfordshire Sarcopenia Study (HSS). Calcified Tissue International, 95:308-316. 10.1007/s00223-014-9894-z 25055749

[9] Kealy J, Murray C, Griffin EW, Lopez-Rodriguez AB, Healy D, Tortorelli LS, et al. (2020). Acute Inflammation Alters Brain Energy Metabolism in Mice and Humans: Role in Suppressed Spontaneous Activity, Impaired Cognition, and Delirium. The Journal of Neuroscience, 40:5681-5696. 10.1523/JNEUROSCI.2876-19.2020 PMC7363463 32513828

[10] Welch C, Majid Z, Greig C, Gladman J, Masud T, Jackson T (2020). Interventions to ameliorate reductions in muscle quantity and function in hospitalised older adults: a systematic review towards acute sarcopenia treatment. Age and Ageing. 10.1093/ageing/afaa209 PMC7936029 33098419

[11] Welch C, Greig CA, Masud T, Pinkney T, Jackson TA (2020). Protocol for understanding acute sarcopenia: a cohort study to characterise changes in muscle quantity and physical function in older adults following hospitalisation. BMC Geriatrics, 20:239. 10.1186/s12877-020-01626-4 PMC7350619 32650734

[12] Wilson DV, Moorey H, Stringer H, Sahbudin I, Filer A, Lord JM, et al. (2019). Bilateral Anterior Thigh Thickness: A New Diagnostic Tool for the Identification of Low Muscle Mass? Journal of the American Medical Directors Association, 20:1247-1253. e1242. 10.1016/j.jamda.2019.04.005 31164257

[13] Sergi G, De Rui M, Veronese N, Bolzetta F, Berton L, Carraro S, et al. (2015). Assessing appendicular skeletal muscle mass with bioelectrical impedance analysis in free-living Caucasian older adults. Clin Nutr, 34:667-673. 10.1016/j.clnu.2014.07.010 25103151

[14] Janssen I, Heymsfield SB, Baumgartner RN, Ross R (2000). Estimation of skeletal muscle mass by bioelectrical impedance analysis. J Appl Physiol (1985), 89:465-471. 10.1152/jappl.2000.89.2.465 10926627

[15] Guralnik JM, Simonsick EM, Ferrucci L, Glynn RJ, Berkman LF, Blazer DG, et al. (1994). A short physical performance battery assessing lower extremity function: association with self-reported disability and prediction of mortality and nursing home admission. J Gerontol, 49:M85-94. 10.1093/geronj/49.2.m85 8126356

[16] American Psychiatric Association. 2013. Diagnostic and Statistical Manual of Mental Disorders.

[17] Tatsuoka C, DeMarco L, Smyth KA, Wilkes S, Howland M, Lerner AJ, et al. (2016). Evaluating PROMIS Physical Function Measures in Older Adults at Risk for Alzheimer's Disease. Gerontology & geriatric medicine, 2:2333721416665502-2333721416665502. 10.1177/2333721416665502 PMC5590694 28913370

[18] Tibshirani R (1996). Regression Shrinkage and Selection via the Lasso. Journal of the Royal Statistical Society. Series B (Methodological), 58:267-288.

[19] Chen X, Gole J, Gore A, He Q, Lu M, Min J, et al. (2020). Non-invasive early detection of cancer four years before conventional diagnosis using a blood test. Nature Communications, 11:3475. 10.1038/s41467-020-17316-z PMC7374162 32694610

[20] Csardi G, Nepusz T (2006). The igraph software package for complex network research. InterJournal, Complex Systems, 1695.

[21] Shannon P, Markiel A, Ozier O, Baliga NS, Wang JT, Ramage D, et al. (2003). Cytoscape: a software environment for integrated models of biomolecular interaction networks. Genome Res, 13:2498-2504. 10.1101/gr.1239303 PMC403769 14597658

[22] Welch C, Greig C, Majid Z, Masud T, Moorey H, Pinkney T, et al. (2021). The feasibility of conducting acute sarcopenia research in hospitalised older patients: a prospective cohort study. European Geriatric Medicine. 10.1007/s41999-021-00565-6 PMC8490139 34608617

[23] Paddon-Jones D, Sheffield-Moore M, Cree MG, Hewlings SJ, Aarsland A, Wolfe RR, et al. (2006). Atrophy and Impaired Muscle Protein Synthesis during Prolonged Inactivity and Stress. The Journal of Clinical Endocrinology & Metabolism, 91:4836-4841. 10.1210/jc.2006-0651 16984982

[24] Bodine SC, Latres E, Baumhueter S, Lai VK-M, Nunez L, Clarke BA, et al. (2001). Identification of Ubiquitin Ligases Required for Skeletal Muscle Atrophy. Science, 294:1704-1708. 10.1126/science.1065874 11679633

[25] Yousuf Y, Datu A, Barnes B, Amini-Nik S, Jeschke MG (2020). Metformin alleviates muscle wasting post-thermal injury by increasing Pax7-positive muscle progenitor cells. Stem Cell Research & Therapy, 11:18. 10.1186/s13287-019-1480-x PMC6950874 31915055

[26] Walton RG, Dungan CM, Long DE, Tuggle SC, Kosmac K, Peck BD, et al. (2019). Metformin blunts muscle hypertrophy in response to progressive resistance exercise training in older adults: A randomized, double-blind, placebo-controlled, multicenter trial: The MASTERS trial. Aging Cell, 18:e13039. 10.1111/acel.13039 PMC6826125 31557380

[27] Watkinson RE, Sutton M, Turner AJ (2021). Ethnic inequalities in health-related quality of life among older adults in England: secondary analysis of a national cross-sectional survey. The Lancet Public Health, 6:e145-e154. 10.1016/S2468-2667(20)30287-5 33516278

[28] Haugen F, Norheim F, Lian H, Wensaas AJ, Dueland S, Berg O, et al. (2010). IL-7 is expressed and secreted by human skeletal muscle cells. Am J Physiol Cell Physiol, 298:C807-816. 10.1152/ajpcell.00094.2009 20089933

[29] Beaudart C, Zaaria M, Pasleau F, Reginster J-Y, Bruyère O (2017). Health Outcomes of Sarcopenia: A Systematic Review and Meta-Analysis. PLOS ONE, 12:e0169548. 10.1371/journal.pone.0169548 PMC5240970 28095426

[30] Vetrano DL, Landi F, Volpato S, Corsonello A, Meloni E, Bernabei R, et al. (2014). Association of Sarcopenia With Short- and Long-term Mortality in Older Adults Admitted to Acute Care Wards: Results From the CRIME Study. The Journals of Gerontology: Series A, 69:1154-1161. 10.1093/gerona/glu034 24744390

[31] Junnila RK, List EO, Berryman DE, Murrey JW, Kopchick JJ (2013). The GH/IGF-1 axis in ageing and longevity. Nat Rev Endocrinol, 9:366-376. 10.1038/nrendo.2013.67 PMC4074016 23591370

[32] Ross R, Miell J, Freeman E, Jones J, Matthews D, Preece M, et al. (1991). Critically ill patients have high basal growth hormone levels with attenuated oscillatory activity associated with low levels of insulin-like growth factor-I. Clinical Endocrinology, 35:47-54. 10.1111/j.1365-2265.1991.tb03495.x 1909610

[33] Fryburg DA, Barrett EJ (1993). Growth hormone acutely stimulates skeletal muscle but not whole-body protein synthesis in humans. Metabolism, 42:1223-1227. 10.1016/0026-0495(93)90285-v 8412780

[34] Mosk CA, van Vugt JLA, de Jonge H, Witjes CD, Buettner S, Ijzermans JN, et al. (2018). Low skeletal muscle mass as a risk factor for postoperative delirium in elderly patients undergoing colorectal cancer surgery. Clinical interventions in aging, 13:2097-2106. 10.2147/CIA.S175945 PMC6205536 30425464

[35] Bellelli G, Zambon A, Volpato S, Abete P, Bianchi L, Bo M, et al. (2018). The association between delirium and sarcopenia in older adult patients admitted to acute geriatrics units: Results from the GLISTEN multicenter observational study. Clinical Nutrition, 37:1498-1504. 10.1016/j.clnu.2017.08.027 28918171

[36] Fisher SR, Goodwin JS, Protas EJ, Kuo Y-F, Graham JE, Ottenbacher KJ, et al. (2011). Ambulatory Activity of Older Adults Hospitalized with Acute Medical Illness. Journal of the American Geriatrics Society, 59:91-95. 10.1111/j.1532-5415.2010.03202.x PMC3133455 21158744

[37] Kortebein P, Ferrando A, Lombeida J, Wolfe R, Evans WJ (2007). Effect of 10 days of bed rest on skeletal muscle in healthy older adults. Jama, 297:1772-1774. 10.1001/jama.297.16.1772-b 17456818

[38] Huang N, Kny M, Riediger F, Busch K, Schmidt S, Luft FC, et al. (2017). Deletion of Nlrp3 protects from inflammation-induced skeletal muscle atrophy. Intensive Care Medicine Experimental, 5:3. 10.1186/s40635-016-0115-0 PMC5241267 28097512

[39] Cape E, Hall RJ, van Munster BC, de Vries A, Howie SEM, Pearson A, et al. (2014). Cerebrospinal fluid markers of neuroinflammation in delirium: a role for interleukin-1β in delirium after hip fracture. Journal of psychosomatic research, 77:219-225. 10.1016/j.jpsychores.2014.06.014 PMC4274366 25124807

[40] Levey AS, Coresh J, Greene T, Stevens LA, Zhang YL, Hendricksen S, et al. (2006). Using Standardized Serum Creatinine Values in the Modification of Diet in Renal Disease Study Equation for Estimating Glomerular Filtration Rate. Annals of Internal Medicine, 145:247-254. 10.7326/0003-4819-145-4-200608150-00004 16908915

[41] Cockcroft DW, Gault MH (1976). Prediction of creatinine clearance from serum creatinine. Nephron, 16:31-41. 10.1159/000180580 1244564

