# Supplementary material for: Establishing Predictors of Acute Sarcopenia: A Proof-Of-Concept Study Utilising Network Analysis
Source: Aging Dis. 2024 Jun 11;16(4):2360–72. doi: 10.14336/AD.2024.0167 (PMC12221406; doi:10.14336/AD.2024.0167)
Supplement: Supplementary file 1 — The Supplementary data can be found online at: www.aginganddisease.org/EN/10.14336/AD.2024.0167. [file AD-16-4-2360-s.pdf]

Establishing Predictors of Acute Sarcopenia: A Proof-Of-Concept Study Utilising Network Analysis

Carly Welch, Laura Bravo, Georgios Gkoutos, Carolyn Greig, Danielle Lewis, Janet Lord, Zeinab Majid, Tahir Masud, Kirsty McGee, Hannah Moorey, Thomas Pinkney, Benjamin Stanley, Thomas Jackson

Establishing biomarkers of acute sarcopenia: a proof-of-concept study utilising network analysis

Contents

Supplementary methods 3

Sample preparation 3

Cortisol ELISA 3

Dehydroepiandrosterone sulfate (DHEA-s) ELISA 3

hsCRP ELISA 3

Growth Hormone (GH) ELISA 4

Insulin-like Growth Factor 1 (IGF-1) ELISA 4

Myostatin ELISA 4

Vitamin D ELISA 5

Human XL cytokine Luminex assay 5

Human obesity premixed ELISA 5

Variables included in analysis 5

Supplementary results 11

SUPPLEMENTARY METHODS

Sample preparation

Blood samples were collected peripherally (or centrally if central access lines were in place as part of routine clinical care) using BD vacutainer tubes. Samples were collected in silicone coated tubes and centrifuged at 3000rpm for 10 minutes within 30-60 minutes of collection for serum separation. Samples were collected in lithium heparin tubes and centrifuged at 1600rpm for 8 minutes for plasma separation. All samples were aliquoted at time of preparation and stored at -80ºC prior to laboratory analysis. Samples were thawed a single time prior to analysis.

Cortisol ELISA

Cortisol was measured using Human Cortisol ELISA Kit (E-EL-0157, Elabscience). Plasma samples were diluted 1:2 using sample diluent. After preparation of reagents and standards, 50µL of standards and diluted samples were pipetted into one well each of the 96T ELISA micro-plates. Samples were pipetted in singlicate across two duplicate plates. Standards were pipetted in duplicate on both plates. Immediately, 50µL of Biotinylated Detection antibody working solution were pipetted into each well. The plates were then covered with a sealer and incubated for 45 minutes at 37ºC. Following this, solution was decanted from each well and 350µL of wash buffer was added to each well. Wash buffer was then decanted and the plate was tapped on absorbent paper. This wash process was repeated a further two times. Next, 100µL of Avidin conjugated to Horseradish Peroxidase (HRP) conjugate working solution was added to each well, and the plates were incubated for 30 minutes at 37ºC. The wash process was then repeated (three further washes), and 90µL of substrate reagent was pipetted into each well. Plates were covered with a plate sealer and incubated for 15 minutes at 37ºC. Finally, 50µL of stop solution were added to each well in the same order as the substrate solution. Optical density was determined immediately using a micro-plate reader set to 450nm. Sample concentrations were calculated from the standard curve using GraphPad Prism 9.2.0, using a four parameter logistic curve model.

Dehydroepiandrosterone sulfate (DHEA-s) ELISA

Dehydroepiandrosterone sulfate (DHEA-s) was measured using Human DHEA-s ELISA Kit (EH2946, FineTest, Wuhan Fine Biotech Co., Ltd.). Plasma samples were diluted 1:2 using sample dilution buffer. Before adding standards and samples, the 96T ELISA micro-plates were washed twice by pipetting 350µL of wash buffer into each well and decanting. After preparation of reagents and standards, 50µL of standards and diluted samples were pipetted into one well each of the plates. Samples were pipetted in singlicate across two duplicate plates. Standards were pipetted in duplicate on both plates. Immediately, 50µL of Biotin-labelled antibody working solution were pipetted into each well. The plates were then covered with a sealer and incubated for 45 minutes at 37ºC. Following this, the wash process was repeated three times. Next, 100µL of HRP-Streptavidin conjugate working solution was added to each well, and the plates were incubated for 30 minutes at 37ºC. The wash process was then repeated (five further washes), and 90µL of substrate reagent was pipetted into each well. Plates were covered with a plate sealer and incubated for 15 minutes at 37ºC. Finally, 50µL of stop solution were added to each well in the same order as the substrate solution. Optical density was determined immediately using a micro-plate reader set to 450nm. Sample concentrations were calculated from the standard curve using GraphPad Prism 9.2.0, using a four parameter logistic curve model.

hsCRP ELISA

High sensitivity C-Reactive Protein (hsCRP) was measured using Human hsCRP ELISA Kit (HK369, HycultBiotech). Standards were diluted 1:100 and serum samples were diluted 1:1000 using sample dilution buffer. Subsequently, 100µL of diluted standards and samples were pipetted into each well of the 96T micro-plates. Samples were pipetted in singlicate across two duplicate plates. Standards were pipetted in duplicate on both plates. The plates were then covered and incubated at room temperature for 30 minutes. Following this, solution was decanted from each well and 350µL of wash buffer was added to each well. Wash buffer was then decanted and the plate was tapped on absorbent paper. This wash process was repeated a further two times. Next, 100µL of conjugate solution was pipetted into each well. Plates were then covered and incubated at room temperature for 30 minutes, following which the washing procedure was repeated. Next,100µL of Chromagen solution was added to each well, then the plates were recovered and incubated for 10 minutes at room temperature. Finally, 50µL of stop solution were added to each well in the same order as the substrate solution. Optical density was determined immediately using a micro-plate reader set to

450nm. Sample concentrations were calculated from the standard curve using GraphPad Prism 9.2.0, using a linear model.

Growth Hormone (GH) ELISA

Growth Hormone (GH) was measured using Human Growth Hormone sandwich ELISA kit (KE00167, Proteintech). Serum samples were diluted 1:2 using sample diluent PT 1-em. After preparation of reagents and standards, 100µL of standards and diluted samples were pipetted into one well each of the 96T ELISA micro-plates. Samples were pipetted in singlicate across two duplicate plates. Standards were pipetted in duplicate on both plates. The plates were then covered with a sealer and incubated for 120 minutes at 37ºC. Following this, solution was decanted from each well and 350µL of wash buffer was added to each well. Wash buffer was then decanted and the plate was tapped on absorbent paper. This wash process was repeated a further three times. Next, 100µL of diluent antibody solution was added to each well, and the plates were incubated for 60 minutes at 37ºC. The wash process was then repeated (four further washes), and 100µL of diluent HRP solution was pipetted into each well. Plates were covered with a plate sealer and incubated for 40 minutes at 37ºC, and the wash process (four further washes) was repeated again. Following this, 100µL of substrate was added to each well and the plate was incubated for a further 15 minutes in the dark at 37ºC. Finally, 100µL of stop solution was added to each well in the same order as the substrate solution. Optical density was determined immediately using a micro-plate reader set to 450nm. Sample concentrations were calculated from the standard curve using GraphPad Prism 9.2.0, using a four parameter logistic curve model.

Insulin-like Growth Factor 1 (IGF-1) ELISA

Insulin-like Growth Factor 1 (IGF-1) was measured using Human IGF-1 ELISA Kit (ELH-IGF1, RayBiotech). Serum samples were diluted 1:2 using diluent buffer. After preparation of reagents and standards, 100µL of standards and diluted samples were pipetted into one well each of the 96T ELISA micro-plates. Samples were pipetted in singlicate across two duplicate plates. Standards were pipetted in duplicate on both plates. The plates were then covered with a sealer and incubated for 150 minutes at room temperature with gentle shaking. Following this, solution was decanted from each well and 300µL of wash buffer was added to each well. Wash buffer was then decanted and the plate was tapped on absorbent paper. This wash process was repeated a further three times. Next, 100µL of biotinylated antibody solution was added to each well, and the plates were incubated for 60 minutes at room temperature with gentle shaking. The wash process was then repeated (four further washes), and 100µL of streptavidin solution was pipetted into each well. Plates were covered with a plate sealer and incubated for 45 minutes at room temperature with gentle shaking, and the wash process (four further washes) was repeated again. Following this, 100µL of substrate reagent was added to each well and the plate was incubated for a further 30 minutes in the dark at room temperature with gentle shaking. Finally, 50µL of stop solution was added to each well in the same order as the substrate solution. Optical density was determined immediately using a micro-plate reader set to 450nm. Sample concentrations were calculated from the standard curve using GraphPad Prism 9.2.0, using a four parameter logistic curve model.

Myostatin ELISA

Myostatin was measured using Human Myostatin ELISA Kit (DL-MSTN-Hu, Dldevelop). Serum samples were diluted 1:2 using diluent buffer. After preparation of reagents and standards, 100µL of standards and diluted samples were pipetted into one well each of the 96T ELISA micro-plates. Samples were pipetted in singlicate across two duplicate plates. Standards were pipetted in duplicate on both plates. The plates were then covered with a sealer and incubated for 120 minutes at 37ºC. Following this, solution was decanted from each well. Next, 100µL of Detection Reagent A working solution was added to each well, and the plates were covered and incubated for 60 minutes at 37ºC. Solution was then decanted from each well and 300µL of wash buffer was added to each well. Wash buffer was then decanted and the plate was tapped on absorbent paper. This wash process was repeated a further two times. Next, 100µL of Detection Reagent B working solution was pipetted into each well. Plates were covered with a plate sealer and incubated for 60 minutes at 37ºC, and the wash process (five further washes) was repeated again. Following this, 90µL of substrate solution was added to each well and the plate was covered and incubated for a further 15 minutes at 37ºC. Finally, 50µL of stop solution was added to each well in the same order as the substrate solution. Optical density was determined immediately using a micro-plate reader set to 450nm. Sample concentrations were calculated from the standard curve using GraphPad Prism 9.2.0, using a four parameter logistic curve model.

Vitamin D ELISA

Total 25-hydroxyvitamin D_2 and 25-hydroxyvitamin D_3 (total 25-OH Vitamin D) was measured using Total 25-OH Vitamin D ELISA Kit (80987, Crystal Chem). The working conjugate was prepared prior to pipetting of samples and standards. Samples were used neat and undiluted in this experiment; 25µL of standards and samples were pipetted into each well of the 96T micro-plates. Samples were pipetted in singlicate across two duplicate plates. Standards were pipetted in duplicate on both plates. Next, 150µL of incubation buffer was added to each wells. The plates were then covered and incubated at room temperature for 60 minutes. Following this, solution was aspirated from wells and wells were washing with wash buffer three times using an automated plate washer (R&D systems). Following this, 150µL of working conjugate solution was pipetted into each well. Plates were then covered and incubated at room temperature for 30 minutes, following which the washing procedure was repeated. Next,150µL of substrate solution was added to each well, then the plates were recovered and incubated for 15 minutes at room temperature. Finally, 50µL of stop solution were added to each well in the same order as the substrate solution. Optical density was determined immediately using a micro-plate reader set to 450nm. Sample concentrations were calculated from the standard curve using GraphPad Prism 9.2.0, using a linear model.

Human XL cytokine Luminex assay

CCL2/JE/MCP-1, CXCL1/GRO alpha/KC/CINC-1, Flt-2 Ligand/FLT3L, IL-1 alpha/IL-1F1, IL-4, IL-7, IL-10, TNF-alpha, CCL3/MIP-1 alpha, CXCL10/IP-10/CRG-2, IFN-gamma, IL-1 beta/IL-1F2, IL-6, IL-8/CXCL8, IL-15, and VEGF were measured using Human XL Cytokine Premixed Luminex Performance Assay Kit (1621325, R&D systems, Bio-techne). Samples were diluted 1:2 with calibrator diluent RD-65. After preparation of reagents and standards, 50µL of standards and diluted samples was pipetted into each well of the 96T micro-titre plates. Samples were pipetted in singlicate across two duplicate plates. Standards were pipetted in duplicate on both plates. Subsequently, 50µL of diluted microparticle cocktail was added to each well of the micro-titre plates. Plates were covered and incubated for 120minutes at room temperature on a horizontal orbital plate shaker set at 800rpm. Following this, solution was aspirated from the wells and the plates were washed with wash buffer three times using an electronic plate washer with a magnetic plate holder (R&D systems). Next, 50µL of diluted Biotin-Antibody cocktail was added to all wells, the plates were covered with a sealer and incubated for 60 minutes at room temperature on the shaker set at 800rpm. The wash process was then repeated. Following this 50µL of diluted Streptavidin-PE was pipetted to all wells. Plates were again covered with a sealer and incubated for 30minutes at room temperature on the shaker at 800rpm. The wash process was again repeated after this. Finally, the microparticles were resuspended by adding 100µL of wash buffer to each well. Plates were incubated on the shaker set at 800rpm for two minutes. Plates were read using a Bio-Rad analyser.

Human obesity premixed ELISA

Resistin and leptin were measured using Human Obesity Premixed Magnetic Luminex Performance Assay Kit (P205396, R&D systems, Bio-techne). Samples were diluted 1:4 with calibrator diluent RD6-46. After preparation of reagents and standards, 50µL of standards and diluted samples was pipetted into each well of the 96T micro-titre plates. Samples were pipetted in singlicate across two duplicate plates. Standards were pipetted in duplicate on both plates. Subsequently, 50µL of diluted microparticle cocktail was added to each well of the micro-titre plates. Plates were covered and incubated for 180minutes at room temperature on a horizontal orbital plate shaker set at 800rpm. Following this, solution was aspirated from the wells and the plates were washed with wash buffer three times using an electronic plate washer with a magnetic plate holder (R&D systems). Next, 50µL of diluted Biotin-Antibody cocktail was added to all wells, the plates were covered with a sealer and incubated for 60 minutes at room temperature on the shaker set at 800rpm. The wash process was then repeated. Following this 50µL of diluted Streptavidin-PE was pipetted to all wells. Plates were again covered with a sealer and incubated for 30minutes at room temperature on the shaker at 800rpm. The wash process was again repeated after this. Finally, the microparticles were resuspended by adding 100µL of wash buffer to each well. Plates were incubated on the shaker set at 800rpm for two minutes. Plates were read using a Bio-Rad analyser.

Variables included in analysis

Supplementary Table 1 shows all the variables which were initially imported for visual inspection of data, and the definitions of these. Biomarkers that did not show differentiation between participants were not included at this stage. Figure S1 demonstrates the frequency of categorical variable datapoints separated by group (elective/ emergency/ medical). Figure S2 and Figure S3 demonstrate the distributions of continuous

variable datapoints separated by group. Table S2 demonstrates the variables that were selected as features within the LASSO and Elastic Net models and subsequent network analysis.

Supplementary Table 1 – Variables initially imported for visual inspection.
Binary variables are shown in blue, continuous variables are shown in green, ordinal variables are shown in orange, and categorical variables are shown in red.

Group	0=emergency surgery, 1=medical, 2=elective surgery
Age	In years
Sex	0=female, 1=male
Ethnicity	0=White British, 1=White Irish, 2=Indian, 3=Arab
Smoking	0=Non-smoker, 1=Ex-smoker, 3=Current smoker
DM	Diabetes Mellitus; 1=yes, 0=no
HF	Heart failure; 1=yes, 0=no
IHD	Ischaemic Heart Disease; 1=yes, 0=no
Stroke	Previous stroke; 1=yes, 0=no
Cancer	Active or recently treated; 1=yes, 0=no
Asthma	Asthma; 1=yes, 0=no
COPD	Chronic Obstructive Pulmonary Disease; 1=yes, 0=no
Anx_Dep	Anxiety/depression; 1=yes, 0=no
Cognitive	Pre-existent cognitive impairment; 1=yes, 0=no
Infection	(Medical patients only): Infection type<br>1=Respiratory, 2=Urinary, 3=Skin, 4=Biliary, 5=COVID-19, 6=Unknown source
Lap_Open	(Surgical patients only): Operation type<br>1=Laparoscopic, 2=Open
Digoxin	Treatment with prior to or during hospitalisation; 1=yes, 0=no
Metformin	Treatment with prior to or during hospitalisation; 1=yes, 0=no
Statin	Treatment with prior to or during hospitalisation; 1=yes, 0=no
Steroids	Treatment with prior to or during hospitalisation; 1=yes, 0=no
ADLs_Baseline	Combined score of Katz (scored out of 6) and Lawton (scored out of 8) ADLs - Baseline
ADLs_V3	Combined score of Katz (scored out of 6) and Lawton (scored out of 8) ADLs - 7 days
ADLs_V4	Combined score of Katz (scored out of 6) and Lawton (scored out of 8) ADLs - 13 weeks
BMI_V1	Body Mass Index - Baseline
BMI_V4	Body Mass Index - 13 weeks
MNA_V1	Mini Nutritional Assessment - Baseline (max score 30)
MNA_V4	Mini Nutritional Assessment - 13 weeks (max score 30)
Nutrition_V1	Categorised MNA Baseline; 0=normal, 1=at risk, 2=malnourished<br>*Calculated using recognised cut-offs from MNA*
Nutrition_V4	Categorised MNA 13 weeks; 0=normal, 1=at risk, 2=malnourished<br>*Calculated using recognised cut-offs from MNA*
Steps_count	Average steps/day in hospital from Fitbit
Steps_900	Categorised from above; 0=less than 900, 1=900 or greater steps/day
Delirium	Delirium during admission; 0=no, 1=yes
LoS	Length of stay in whole days
Hospital_Total	Total days in acute hospital from baseline assessment to 13 weeks (including readmissions)
Death_IP	Death during admission; 0=no, 1=yes
TBW_V1	Total body water (bioelectrical impedance analysis) - baseline
TBW_V2	Total body water (bioelectrical impedance analysis) - within 48 hours of surgery (elective only)
TBW_V3	Total body water (bioelectrical impedance analysis) - 7 days post-operative/admission
TBW_V4	Total body water (bioelectrical impedance analysis) - 13 weeks post-operative/admission
TBW%_V1	TBW as % of total weight (bioelectrical impedance analysis) - baseline
TBW%_V2	TBW as % of total weight (bioelectrical impedance analysis) - 48 hours (elective only)
TBW%_V3	TBW as % of total weight (bioelectrical impedance analysis) - 7 days
TBW%_V4	TBW as % of total weight(bioelectrical impedance analysis) - 13 weeks
ECW_V1	Extracellular water (bioelectrical impedance analysis) - baseline
ECW_V2	Extracellular water(bioelectrical impedance analysis) - 48 hours (elective only)
ECW_V3	Extracellular water (bioelectrical impedance analysis) - 7 days
ECW_V4	Extracellular water (bioelectrical impedance analysis) - 13 weeks
ECW%_V1	ECW as % of total weight (bioelectrical impedance analysis) - baseline
ECW%_V2	ECW as % of total weight (bioelectrical impedance analysis) - 48 hours (elective only)
ECW%_V3	ECW as % of total weight (bioelectrical impedance analysis) - 7 days
ECW%_V4	ECW as % of total weight (bioelectrical impedance analysis) - 13 weeks

ICW_V1	Intracellular water (bioelectrical impedance analysis) - baseline
ICW_V2	Intracellular water(bioelectrical impedance analysis) - 48 hours (elective only)
ICW_V3	Intracellular water (bioelectrical impedance analysis) - 7 days
ICW_V4	Intracellular water (bioelectrical impedance analysis) - 13 weeks
ICW%_V1	ICW as % of total weight (bioelectrical impedance analysis) - baseline
ICW%_V2	ICW as % of total weight (bioelectrical impedance analysis) - 48 hours (elective only)
ICW%_V3	ICW as % of total weight (bioelectrical impedance analysis) - 7 days
ICW%_V4	ICW as % of total weight (bioelectrical impedance analysis) - 13 weeks
PROMIS_Baseline	Patient reported outcome measures information system physical function Z score - Baseline
PROMIS_V3	Patient reported outcome measures information system physical function Z score - 7 days
PROMIS_V4	Patient reported outcome measures information system physical function Z score - 13 weeks
BATT_V1	Bilateral Anterior Thigh Thickness (ultrasound) - Baseline
BATT_V2	Bilateral Anterior Thigh Thickness (ultrasound) - 48 hours (elective only)
BATT_V3	Bilateral Anterior Thigh Thickness (ultrasound) - 7 days
BATT_V4	Bilateral Anterior Thigh Thickness (ultrasound) - 13 weeks
BATTSCR_V1	BATT: Subcutaneous Ratio (ultrasound) - Baseline
BATTSCR_V2	BATT: Subcutaneous Ratio (ultrasound) - 48 hours (elective only)
BATTSCR_V3	BATT: Subcutaneous Ratio (ultrasound) - 7 days
BATTSCR_V4	BATT: Subcutaneous Ratio (ultrasound) - 13 weeks
Echo_V1	Rectus femoris echogenicity (ultrasound gray scale) - Baseline
Echo_V2	Rectus femoris echogenicity (ultrasound gray scale) - 48 hours (elective only)
Echo_V3	Rectus femoris echogenicity (ultrasound gray scale) - 7 days
Echo_V4	Rectus femoris echogenicity (ultrasound gray scale) - 13 weeks
SMMSergi_V1	Skeletal Muscle Mass (Sergi equation) (Bioelectrical impedance analysis) - Baseline
SMMSergi_V2	Skeletal Muscle Mass (Sergi equation) (Bioelectrical impedance analysis) - 48 hours (elective)
SMMSergi_V3	Skeletal Muscle Mass (Sergi equation) (Bioelectrical impedance analysis) - 7 days
SMMSergi_V4	Skeletal Muscle Mass (Sergi equation) (Bioelectrical impedance analysis) - 13 weeks
SMMJanssen_V1	Skeletal Muscle Mass (Janssen equation) (Bioelectrical impedance analysis) - Baseline
SMMJanssen_V2	Skeletal Muscle Mass (Janssen equation) (Bioelectrical impedance analysis) - 48 hours (elective)
SMMJanssen_V3	Skeletal Muscle Mass (Janssen equation) (Bioelectrical impedance analysis) - 7 days
SMMJanssen_V4	Skeletal Muscle Mass (Janssen equation) (Bioelectrical impedance analysis) - 13 weeks
PA_V1	Phase angle (Bioelectrical impedance analysis) - Baseline
PA_V2	Phase angle (Bioelectrical impedance analysis) - 48 hours (elective)
PA_V3	Phase angle (Bioelectrical impedance analysis) - 7 days
PA_V4	Phase angle (Bioelectrical impedance analysis) - 13 weeks
HGS_V1	Handgrip strength - Baseline
HGS_V2	Handgrip strength - 48 hours (elective)
HGS_V3	Handgrip strength - 7 days
HGS_V4	Handgrip strength - 13 weeks
WS_V1	Gait (walking) speed - Baseline
WS_V3	Gait (walking) speed - 7 days
WS_V4	Gait (walking) speed - 13 weeks
SPPB_V1	Short Physical Perfomance Battery - Baseline<br>*Score 0 to 12, derived from continuous variables, of which gait speed is one of, and normally analysed as continuous*
SPPB_V3	Short Physical Perfomance Battery - 7 days
SPPB_V4	Short Physcial Performance Battery - 13 weeks
V1_CFS	Clinical Frailty Scale - Baseline<br>*Scored 1 (very fit) to 8 (very severely frail)*
V3_CFS	Clinical Frailty Scale - 7 days
V4_CFS	Clinical Frailty Scale - 13 weeks
V1_CFS_Frail	Frailty defined by CFS - Baseline; 0=no, 1=yes<br>*Frailty defined as CFS greater than or equal to 5*
V3_CFS_Frail	Frailty defined by CFS - 7 days; 0=no, 1=yes
V4_CFS_Frail	Frailty defined by CFS - 13 weeks; 0=no, 1=yes
V1_FI	Frailty index - Baseline<br>*Derived from 36 separate variables - count of these divided by 36 gives index between 0 and 1*
V3_FI	Frailty index - 7 days
V4_FI	Frailty index - 13 weeks
V1_FI_Frail	Frailty defined by FI - Baseline; 0=no, 1=yes<br>*Frailty defined by FI greater than or equal to 0.25*
V3_FI_Frail	Frailty defined by FI - 7 days; 0=no, 1=yes
V4_FI_Frail	Frailty defined by FI - 13 weeks; 0=no, 1=yes

V1_Fried_Frail	Frailty defined by Fried - Baseline; 0=no, 1=yes<br>*Frailty defined as three or more of: low handgrip strength (defined cut-offs), low gait speed (defined cut-offs), weight loss >4.5kg/5% over last year, self-reported exhaustion, low physical activity*
V3_Fried_Frail	Frailty defined by FI - 7 days; 0=no, 1=yes
V4_Fried_Frail	Frailty defined by FI - 13 weeks; 0=no, 1=yes
V1_Sarc_Any	Sarcopenia - Baseline; 0=no, 1=yes<br>*Defined as 1) handgrip strength below recognised cut-off AND 2) BATT below recognised or cut-off OR SMMSergi below recognised cut-off*
V3_Sarc_Any	Sarcopenia - 7 days; 0=no, 1=yes
V4_Sarc_Any	Sarcopenia - 13 weeks; 0=no, 1 yes
Hb_V0	Haemoglobin - Preoperative (routine clinical bloods)
Hb_V1	Haemoglobin - within 48 hours of admission or surgery (routine clinical bloods)
Hb_V3	Haemoglobin - 7 days (routine clinical bloods)
Creat_V0	Creatinine - Preoperative (routine clinical bloods)
Creat_V1	Creatinine - within 48 hours of admission or surgery (routine clinical bloods)
Creat_V3	Creatinine - 7 days (routine clinical bloods)
eGFR_V0	Glomerular Filtration Rate - Preoperative (routine clinical bloods)
eGFR_V1	Glomerular Filtration Rate - within 48 hours of admission or surgery (routine clinical bloods)
eGFR_V3	Glomerular Filtration Rate - 7 days (routine clinical bloods)
CRP_V0	C-Reactive Protein - preoperative (from hsCRP ELISA - Elective, or routine clinical bloods - emergency surgery
CRP_V1	C-reactive Protein - within 48 hours of admission or surgery (routine clinical bloods)
CRP_V3	C-reactive Protein - 7 days (routine clinical bloods)
Alb_V0	Albumin - Preoperative (routine clinical bloods)
Alb_V1	Albumin - within 48 hours of admission or surgery (routine clinical bloods)
Alb_V3	Albumin - 7 days (routine clinical bloods)
WCC_V0	White cell count - Preoperative (routine clinical bloods)
WCC_V1	White cell count - Within 48 hours of admission or surgery (routine clinical bloods)
WCC_V3	White cell count - 7 days (routine clinical bloods)
Neu_V0	Neutrophil count - Preoperative (routine clinical bloods)
Neu_V1	Neutrophil count - Within 48 hours of admission or surgery (routine clinical bloods)
Neu_V3	Neutrophil count - 7 days (routine clinical bloods)
Lym_V0	Lymphocyte count - Preoperative (routine clinical bloods)
Lym_V1	Lymphocyte count - Within 48 hours of admission or surgery (routine clinical bloods)
Lym_V3	Lymphocyte count - 7 days (routine clinical bloods)
Myostatin_V0	Myostatin - Preoperative (ELISA)
Myostatin_V1	Myostatin - Within 48 hours of admission or surgery (ELISA)
Cortisol_V0	Cortisol - Preoperative (ELISA)
Cortisol_V1	Cortisol - Within 48 hours of admission or surgery (ELISA)
DHEAS_V0	Dehydroepiandrosterone sulfate - Preoperative (ELISA)
DHEAS_V1	Dehydroepiandrosterone sulfate - Within 48 hours of admission or surgery (ELISA)
IGF-1_V0	Insulin-like growth factor 1 - Preoperative (ELISA)
IGF-1_V1	Insulin-like growth factor 1 - Within 48 hours of admission or surgery (ELISA)
GH_V0	Growth Hormone - Preoperative (ELISA)
GH_V1	Growth Hormone - Within 48 hours of admission or surgery (ELISA)
VitD_V0	25-OH Vitamin D - Preoperative (ELISA)
VitD_V1	25-OH Vitamin D - Within 48 hours of admission or surgery (ELISA)
CCL2_V0	Luminex
CCL2_V1	Luminex
CXCL10_V0	Luminex
CXCL10_V1	Luminex
IL-1a_V0	Luminex
IL-1a_V1	Luminex
IL-6_V0	Luminex
IL-6_V1	Luminex
IL-10_V0	Luminex
IL-10_V1	Luminex
VEGF_V0	Luminex
VEGF_V1	Luminex
IL-1b_V0	Luminex
IL-1b_V1	Luminex
IL-7_V0	Luminex
IL-7_V1	Luminex

IL-15_V0	Luminex
IL-15_V1	Luminex
CXCL1_V0	Luminex
CXCL1_V1	Luminex
IL-8_V0	Luminex
IL-8_V1	Luminex
TNFa_V0	Luminex
TNFa_V1	Luminex
Leptin_V0	Luminex
Leptin_V1	Luminex
Resistin_V0	Luminex
Resistin_V1	Luminex

Supplementary Table 2 – Features included in analysis.
Features that were included if fewer than 30% missing variables were present are shown in orange. Features that were specifically considered in the second analysis focusing on participants where these variables were present are highlighted in gold.

Group	0=emergency surgery, 1=medical, 2=elective surgery
Age	In years
Sex	0=female, 1=male
Ethnicity	0=White British, 1=White Irish, 2=Indian, 3=Arab
Smoking	0=Non-smoker, 1=Ex-smoker, 3=Current smoker
DM	Diabetes Mellitus; 1=yes, 0=no
HF	Heart failure; 1=yes, 0=no
IHD	Ischaemic Heart Disease; 1=yes, 0=no
Stroke	Previous stroke; 1=yes, 0=no
Cancer	Active or recently treated; 1=yes, 0=no
Asthma	Asthma; 1=yes, 0=no
COPD	Chronic Obstructive Pulmonary Disease; 1=yes, 0=no
Anx_Dep	Anxiety/depression; 1=yes, 0=no
Cognitive	Pre-existent cognitive impairment; 1=yes, 0=no
Infection	(Medical patients only): Infection type<br>1=Respiratory, 2=Urinary, 3=Skin, 4=Biliary, 5=COVID-19, 6=Unknown source
Lap_Open	(Surgical patients only): Operation type<br>1=Laparoscopic, 2=Open
Digoxin	Treatment with prior to or during hospitalisation; 1=yes, 0=no
Metformin	Treatment with prior to or during hospitalisation; 1=yes, 0=no
Statin	Treatment with prior to or during hospitalisation; 1=yes, 0=no
Steroids	Treatment with prior to or during hospitalisation; 1=yes, 0=no
BMI_V1	Body Mass Index - Baseline
BMI_V4	Body Mass Index - 13 weeks
MNA_V1	Mini Nutritional Assessment - Baseline (max score 30)
MNA_V4	Mini Nutritional Assessment - 13 weeks (max score 30)
Nutrition_V1	Categorised MNA Baseline; 0=normal, 1=at risk, 2=malnourished<br>*Calculated using recognised cut-offs from MNA*
Nutrition_V4	Categorised MNA 13 weeks; 0=normal, 1=at risk, 2=malnourished<br>*Calculated using recognised cut-offs from MNA*
Steps_count	Average steps/day in hospital from Fitbit

Steps_900	Categorised from above; 0=less than 900, 1=900 or greater steps/day
Delirium	Delirium during admission; 0=no, 1=yes
LoS	Length of stay in whole days
Hospital_Total	Total days in acute hospital from baseline assessment to 13 weeks (including readmissions)
Hb_V0	Haemoglobin - Preoperative (routine clinical bloods)
Hb_V1	Haemoglobin - within 48 hours of admission or surgery (routine clinical bloods)
Hb_V3	Haemoglobin - 7 days (routine clinical bloods)
Creat_V0	Creatinine - Preoperative (routine clinical bloods)
Creat_V1	Creatinine - within 48 hours of admission or surgery (routine clinical bloods)
Creat_V3	Creatinine - 7 days (routine clinical bloods)
eGFR_V0	Glomerular Filtration Rate - Preoperative (routine clinical bloods)
eGFR_V1	Glomerular Filtration Rate - within 48 hours of admission or surgery (routine clinical bloods)
eGFR_V3	Glomerular Filtration Rate - 7 days (routine clinical bloods)
CRP_V0	C-Reactive Protein - preoperative (from hsCRP ELISA - Elective, or routine clinical bloods - emergency surgery
CRP_V1	C-reactive Protein - within 48 hours of admission or surgery (routine clinical bloods)
CRP_V3	C-reactive Protein - 7 days (routine clinical bloods)
Alb_V0	Albumin - Preoperative (routine clinical bloods)
Alb_V1	Albumin - within 48 hours of admission or surgery (routine clinical bloods)
Alb_V3	Albumin - 7 days (routine clinical bloods)
WCC_V0	White cell count - Preoperative (routine clinical bloods)
WCC_V1	White cell count - Within 48 hours of admission or surgery (routine clinical bloods)
WCC_V3	White cell count - 7 days (routine clinical bloods)
Neu_V0	Neutrophil count - Preoperative (routine clinical bloods)
Neu_V1	Neutrophil count - Within 48 hours of admission or surgery (routine clinical bloods)
Neu_V3	Neutrophil count - 7 days (routine clinical bloods)
Lym_V0	Lymphocyte count - Preoperative (routine clinical bloods)
Lym_V1	Lymphocyte count - Within 48 hours of admission or surgery (routine clinical bloods)
Lym_V3	Lymphocyte count - 7 days (routine clinical bloods)
Myostatin_V0	Myostatin - Preoperative (ELISA)
Myostatin_V1	Myostatin - Within 48 hours of admission or surgery (ELISA)
Cortisol_V0	Cortisol - Preoperative (ELISA)
Cortisol_V1	Cortisol - Within 48 hours of admission or surgery (ELISA)
DHEAS_V0	Dehydroepiandrosterone sulfate - Preoperative (ELISA)
DHEAS_V1	Dehydroepiandrosterone sulfate - Within 48 hours of admission or surgery (ELISA)
IGF-1_V0	Insulin-like growth factor 1 - Preoperative (ELISA)
IGF-1_V1	Insulin-like growth factor 1 - Within 48 hours of admission or surgery (ELISA)
GH_V0	Growth Hormone - Preoperative (ELISA)
GH_V1	Growth Hormone - Within 48 hours of admission or surgery (ELISA)
VitD_V0	25-OH Vitamin D - Preoperative (ELISA)
VitD_V1	25-OH Vitamin D - Within 48 hours of admission or surgery (ELISA)
CCL2_V0	Luminex
CCL2_V1	Luminex
CXCL10_V0	Luminex
CXCL10_V1	Luminex
IL-1a_V0	Luminex

IL-1a_V1	Luminex
IL-6_V0	Luminex
IL-6_V1	Luminex
IL-10_V0	Luminex
IL-10_V1	Luminex
VEGF_V0	Luminex
VEGF_V1	Luminex
IL-1b_V0	Luminex
IL-1b_V1	Luminex
IL-7_V0	Luminex
IL-7_V1	Luminex
IL-15_V0	Luminex
IL-15_V1	Luminex
CXCL1_V0	Luminex
CXCL1_V1	Luminex
IL-8_V0	Luminex
IL-8_V1	Luminex
TNFa_V0	Luminex
TNFa_V1	Luminex
Leptin_V0	Luminex
Leptin_V1	Luminex
Resistin_V0	Luminex
Resistin_V1	Luminex

Supplementary Table 3 – Mean and median concentrations of systemic biomarkers separated by sarcopenia status

		Baseline		p value	7 days		p value
		No sarcopenia	Sarcopenia		No sarcopenia	Sarcopenia	
Hb (g/L) *Mean*	Preop	120.9 (7.0)	126.5 (7.0)	0.605[a]	123.5 (28.3)	121.3 (22.0)	0.849[a]
	Acute	113.7 (21.9)	114.5 (16.9)	0.850[a]	113.2 (18.4)	110.5 (17.2)	0.561[a]
WCC (10^9/L) *Median*	Preop	7.0 (6.1 – 8.4)	7.3 (6.15 – 8.5)	0.987[b]	6.85 (6.45 – 8.2)	7.7 (6.15 – 9.65)	0.778[b]
	Acute	10.6 (7.7 – 13.9)	9.5 (8.1 – 13.4)	0.976[b]	9.45 (8.05 – 13.3)	10.85 (8.2 – 14.3)	0.568[b]
Neutrophils (10^9/L) *Median*	Preop	4.75 (4.3 – 6.1)	4.7 (3.45 – 6.15)	0.801[b]	4.45 (3.9 – 5.65)	4.95 (3.45 – 6.85)	0.779[b]
	Acute	7.9 (6.1 – 11.7)	7.9 (6.0 – 12.2)	0.976[b]	7.4 (6.15 – 11.4)	8.65 (5.8 – 12.3)	0.821[b]
Lymphocytes (10^9/L) *Median*	Preop	1.3 (0.9 – 2.0)	1.45 (1.25 – 1.75)	0.709[b]	1.5 (1.2 – 2.5)	1.45 (1.2 – 1.9)	0.820[b]
	Acute	0.7 (0.5 – 1.1)	0.8 (0.5 – 1.3)	0.229[b]	0.7 (0.5 – 1.1)	0.8 (0.6 – 1.3)	0.224[b]
Albumin (g/L) *Mean*	Preop	38.6 (3.4)	35.9 (1.3)	0.096[a]	39.3 (3.9)	37.1 (3.4)	0.214[a]
	Acute	29.6 (5.6)	28.8 (5.4)	0.537[a]	30.1 (5.7)	29.2 (5.5)	0.509[a]
Creatinine (micromol/L) *Median*	Preop	89 (81 – 101)	74 (67 – 85)	0.170[b]	89 (77.5 – 89.5)	82.5 (69 – 102)	0.836[b]
	Acute	92 (72 – 120)	81.5 (61 – 111)	0.180[b]	87 (78 – 132)	84.5 (61 – 111)	0.275[b]
eGFR *Median*	Preop	76 (41 – 81)	67 (60 – 89.5)	0.731[b]	71 (53 – 76)	65 (53 – 90)	0.678[b]
	Acute	60 (46 – 81)	68.5 (48 – 90)	0.409[b]	60 (46 – 81)	71 (47 – 90)	0.545[b]

hsCRP (mg/L) *Median*	Preop	8.17 (5.74 – 9.18)	4.72 (1.01 – 8.07)	0.170[b]	7.01 (1.92 – 9.36)	7.82 (1.63 – 9.00)	0.902[b]
CRP (mg/L) *Median*	Acute	99 (75 – 158)	113 (78 – 194)	0.272[b]	123 (90.5 – 174.5)	109 (67 – 194)	0.568[b]
Myostatin (ng/mL) *Mean*	Preop	20.8 (15.5)	22.7 (9.9)	0.754[a]	15.1 (10.1)	26.1 (14.1)	0.063[a]
	Acute	26.2 (20.2)	29.1 (3.0)	0.565[a]	23.7 (18.1)	28.6 (15.4)	0.332[a]
Cortisol (ng/mL) *Median*	Preop	57.5 (35.4 – 75.3)	100.9 (44.5 – 190.5)	0.238[b]	68.9 (36.5 – 74.9)	73.0 (38.5 – 167.8)	0.345[b]
	Acute	85.1 (75.0 – 123.3)	78.9 (78.0 – 104.8)	0.915[b]	78.5 (90.5 – 115.3)	90.7 (67.0 – 111.6)	0.643[b]
DHEA-s (ng/mL) *Median*	Preop	192.2 (176.9 – 311.1)	160.9 (150.6 – 230.1)	0.192[b]	192.9 (178.5 – 288.7)	193.1 (150.6 – 357.4)	0.862[b]
	Acute	291.7 (159.7 – 426.0)	219.7 (95.3 – 503.0)	0.307[b]	255.6 (159.7 – 392.2)	219.7 (95.3 – 581.5)	0.831[b]
IGF-1 (ng/mL) *Median*	Preop	2.92 (1.72 – 5.60)	2.22 (0.84 – 4.65)	0.566[b]	1.51 (0.62 – 4.65)	2.75 (2.24 – 3.62)	0.607[b]
	Acute	1.83 (0.78 – 8.58)	2.87 (0.37 – 12.2)	0.825[b]	1.56 (0.69 – 18.3)	2.41 (0.37 – 6.57)	0.771[b]
Growth Hormone (pg/mL) *Median*	Preop	406.5 (118.9 – 1643.8)	234.2 (81.4 – 491.7)	0.443[b]	1087.1 (217.8 – 2116.0)	164.9 (102.2 – 491.7)	0.122[b]
	Acute	1327.1 (478.4 – 2375.1)	812.8 (514.3 – 2076.5)	0.492[b]	1284.3 (478.4 – 3231.8)	985.4 (514.3 – 1463.0)	0.545[b]
Vitamin D (ng/mL) *Median*	Preop	19.3 (6.6 – 23.7)	20.7 (15.4 – 24.8)	0.662[b]	20.9 (1.8 – 23.2)	18.3 (15.4 – 24.8)	0.371[b]
	Acute	14.4 (3.5 – 22.9)	19.1 (3.1 – 24.0)	0.602[b]	13.4 (3.2 – 22.1)	19.3 (4.6 – 24.1)	0.520[b]
CCL2 (pg/mL) *Median*	Preop	223.6 (186.4 – 289.2)	346.1 (188.3 – 540.5)	0.166[b]	228.4 (186.4 – 289.2)	309.3 (183.8 – 520.2)	0.370[b]
	Acute	252.9 (157.2 – 445.3)	291.8 (159.9 – 355.6)	0.931[b]	226.5 (149.3 – 355.6)	303.8 (166.8 – 421.2)	0.217[b]
CXCL10 (pg/mL) *Median*	Preop	5.08 (2.22 – 53.3)	51.6 (17.9 – 125.2)	0.093[b]	24.1 (6.1 – 65.4)	42.2 (10.1 – 112.1)	0.755[b]
	Acute	2.4 (2.2 – 39.4)	12.6 (4.6 – 59.5)	0.242[b]	10.6 (2.2 – 42.3)	8.4 (2.2 – 59.5)	0.929[b]
IL-1a (pg/mL) *Median*	Preop	12.2 (10.7 – 12.2)	13.7 (12.2 – 15.8)	0.154[b]	12.2 (12.2 – 12.2)	13.7 (10.7 – 13.7)	0.719[b]
	Acute	10.7 (9.2 – 13.7)	12.6 (10.7 – 14.4)	0.176[b]	10.7 (9.2 – 13.7)	12.2 (10.7 - 13.7)	0.720[b]
IL-6 (pg/mL) *Median*	Preop	9.4 (7.0 – 10.4)	7.5 (4.0 – 103.4)	0.203[b]	8.4 (7.0 – 10.4)	8.9 (5.1 – 12.4)	0.952[b]
	Acute	88.8 (21.4 – 155.8)	37.6 (23.0 – 103.4)	0.170[b]	69.2 (27.8 – 130.6)	37.6 (25.2 – 106.0)	0.397[b]
IL-10 (pg/mL) *Median*	Preop	29.9 (16.0 – 58.7)	39.4 (23.1 – 301.9)	0.598[b]	44.4 (16.1 – 107.4)	37.0 (27.5 – 51.4)	0.976[b]
	Acute	32.3 (22.9 – 52.4)	66.0 (28.7 – 194.1)	0.094[b]	51.4 (22.9 – 80.8)	41.7 (25.2 – 80.8)	0.970[b]
VEGF (pg/mL) *Median*	Preop	109.5 (80.6 – 163.4)	120.6 (78.8 – 190.0)	0.973[b]	101.0 (80.6 – 155.6)	140.1 (83.2 – 209.3)	0.370[b]
	Acute	262.2 (126.0 – 382.1)	273.2 (158.9 – 459.5)	0.561[b]	270.3 (132.1 – 349.5)	239.2 (137.9 – 486.4)	0.857[b]
IL-7 (pg/mL) *Median*	Preop	6.32 (4.93 – 6.93)	7.44 (5.52 – 8.16)	0.132[b]	6.52 (6.12 – 6.93)	6.93 (5.32 – 8.57)	0.399[b]
	Acute	6.52 (5.72 – 7.75)	8.78 (6.52 – 10.25)	0.014*[b]	6.52 (6.12 – 7.75)	8.42 (6.32 – 10.78)	0.063[b]
IL-15 (pg/mL) *Median*	Preop	3.33 (2.66 – 3.56)	3.33 (3.22 – 3.45)	0.722[b]	3.33 (3.33 – 3.79)	3.33 (2.88 – 5.03)	0.392[b]
	Acute	3.78 (3.1 – 4.5)	4.25 (3.33 – 5.0)	0.133[b]	3.79 (3.1 – 4.25)	4.14 (3.33 – 5.03)	0.278[b]
CXCL1 (pg/mL) *Median*	Preop	69.4 (61.7 – 101.3)	76.2 (35.4 – 145.1)	0.829[b]	67.9 (61.7 – 99.0)	84.5 (47.6 – 139.0)	0.515[b]
	Acute	97.8 (58.1 – 157.8)	110.0 (70.9 – 161.1)	0.465[b]	98.4 (64.9 – 138.4)	103.3 (65.22 – 148.8)	0.713[b]
IL-1b (pg/mL) *Median*	Preop	2.04 (1.29 – 3.52)	2.23 (2.04 – 3.15)	0.477[b]	2.04 (1.29 – 2.04)	2.41 (2.04 – 3.52)	0.050*[b]

	Acute	2.04 (1.29 – 2.78)	2.04 (1.67 – 2.78)	0.461[b]	1.67 (1.29 – 2.78)	2.04 (2.035 – 2.78)	0.142[b]
IL-8 (pg/mL) *Median*	Preop	11.0 (6.7 – 16.3)	21.7 (10.5 – 30.4)	0.110[b]	12.1 (10.0 – 17.8)	16.3 (9.0 – 29.4)	0.656[b]
	Acute	11.2 (6.3 – 23.2)	13.2 (10.5 – 21.3)	0.280[b]	10.2 (6.3 – 18.0)	13.2 (10.3 – 29.3)	0.144[b]
TNF-α (pg/mL) *Median*	Preop	11.2 (8.8 – 14.8)	16.0 (8.8 – 22.7)	0.254[b]	11.6 (8.8 – 17.3)	15.0 (8.8 – 17.7)	0.719[b]
	Acute	14.8 (10.0 – 16.9)	13.6 (11.2 – 18.5)	0.668[b]	15.2 (10.4 – 18.5)	12.8 (10.8 – 18.1)	0.765[b]
Leptin (pg/mL) *Median*	Preop	13914 (9619 – 22260)	12328 (6340 – 32851)	0.881[b]	16368 (9940 – 17482)	16979 (9292 – 26582)	0.719[b]
	Acute	13871 (5510 – 32766)	4598 (2109 – 17928)	0.668[b]	14262 (5716 – 28725)	5711 (2251 – 21709)	0.765[b]
Resistin (pg/mL) *Median*	Preop	8873 (7130 – 13602)	9616 (7725 – 13536)	0.788[b]	7949 (7068 – 13941)	9952 (8468 – 12564)	0.719[b]
	Acute	20625 (11828 – 28384)	17165 (11356 – 27014)	0.668[b]	20970 (13144 – 41934)	15394 (11167 – 23891)	0.765[b]

[a]Unpaired t-test; [b]Wilcoxon rank-sum test

Hb=Haemoglobin; WCC=White Cell Count; eGFR=estimated Glomerular Filtration Rate; hsCRP=High sensitivity C-Reactive Protein; CRP=C-Reactive Protein; DHEA-s=Dehydroepiandrosterone sulfate; IGF-1=Insulin-like Growth Factor 1; CCL2=Chemokine (C-C motif) ligand 2; CXCL10=Chemokine (C-X-C motif) ligand 10; IL-1a=Interleukin 1a; IL-6=Interleukin 6; IL-10=Interleukin 10; VEGF=Vascular Endothelial Growth Factor; IL-7=Interleukin 7; IL-15; Interleukin 15; CXCL1=Chemokine (C-X-C motif) ligand 1; IL-1b=Interleukin 1b; IL-8=Interleukin 8; TNF-α=Tumour Necrosis Factor Alpha

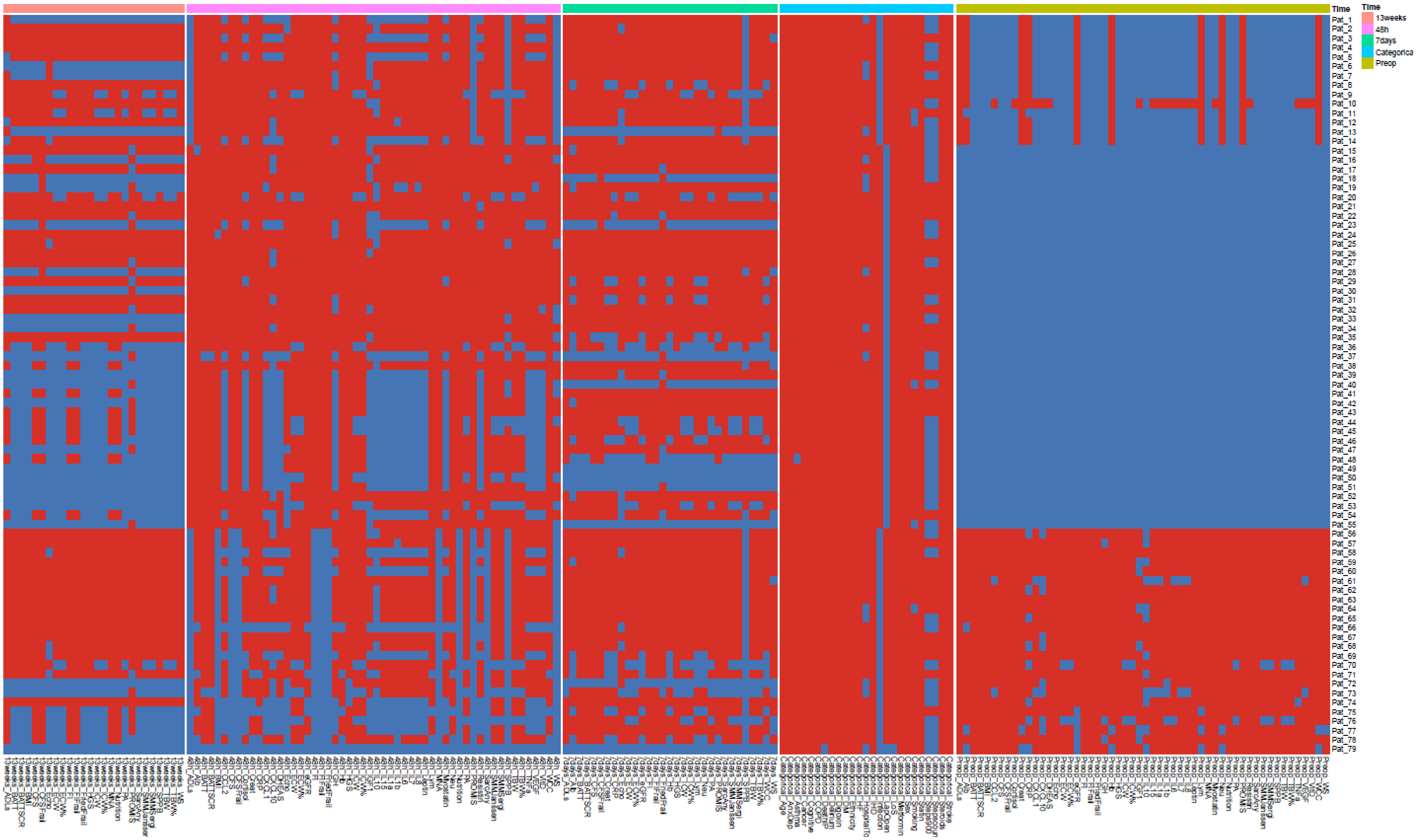

Supplementary Figure 1 – Heatmap showing missing variables.

Missing variables are shown in blue, variables that were available are shown in red.

SUPPLEMENTARY DATA

SUPPLEMENTARY RESULTS

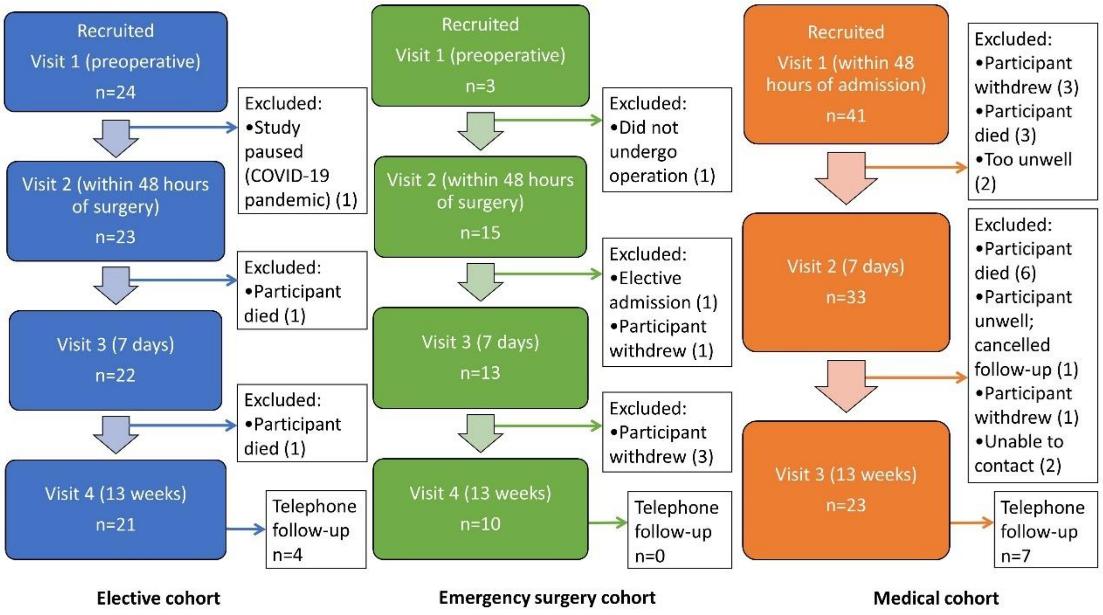

Supplementary Figure 2 – Recruitment flowchart for participants separated by group

Supplementary Table 4 – Beta coefficients derived from LASSO and Elastic Net models for outcomes at timepoints, with specific focus on participants with additional systemic biomarkers available. Results are adjusted for baseline sarcopenia status. Square brackets denote confidence intervals for coefficients. Curved brackets denote the number of models that the association was encountered within, and the number of models that the association was tested within. The timing of the individual variables and outcomes tested are denoted in the first column and row respectively. Variables without timing specified in the first column are constants. The separate timing (second) column refers to the timing of other variables that the associated was tested against. Non-significant associations are not shown.

	Timing	BATT (7 days)	BATT (13 weeks)	SMMSergi (7 days)	Echo (7 days)	Sarc (7 days)	Sarc (13 weeks)
ADLs (Preop)	Preop	0.16 [0.1, 0.22] (8/23)					
Anxiety/ Depression	Preop				-0.6 [-0.83, -0.36] (8/23)		
Asthma	Preop						-0.9 [-1.63, -0.17] (3/11)
BATT (48 hours)	48 hours					-0.12 [-0.18, -0.06] (21/35)	
BMI (Preop)	Preop		0.21 [0.14, 0.27] (9/23)	0.13 [0.11, 0.15] (17/23)			
Cancer	Preop						-0.24 [-0.32, -0.17]

© 2024. Welch C et al. Published online at http://www.aginganddisease.org/EN/10.14336/AD.2024.0167

							(6/11)
CCL2 (Preop)	Preop			-0.17 [-0.26, -0.08] (6/23)			
COPD	Preop				0.41 [0.29, 0.54] (8/23)		
	48 hours	-0.43 [-0.46, -0.41] (50/50)		-0.37 [-0.39, -0.34] (50/50)	0.22 [0.18, 0.25] (44/50)	0.6 [0.48, 0.71] (34/35)	
Cortisol (Preop)	Preop	-0.18 [-0.24, -0.11] (11/23)		-0.08 [-0.12, -0.04] (9/23)			
Creatinine (Preop)	Preop	0.24 [0.12, 0.37] (15/23)	0.21 [0.01, 0.42] (5/23)	0.35 [0.3, 0.39] (19/23)	-0.16 [-0.23, -0.08] (9/23)		
Digoxin	48 hours	-0.31 [-0.35, -0.28] (42/50)					
Diabetes Mellitus	Preop		0.3 [0.13, 0.48] (9/23)	0.15 [0.02, 0.27] (7/23)			
	48 hours	0.32 [0.28, 0.36] (49/50)					
eGFR (Preop)	Preop				0.15 [0.1, 0.2] (9/23)		
eGFR (48 hours)	48 hours			-0.08 [-0.1, -0.07] (46/50)			
GH (Preop)	Preop		0.27 [0.18, 0.35] (5/23)				
HGS (Preop)	Preop			0.1 [0.06, 0.13] (15/23)			
Handgrip strength (48 hours)	48 hours	0.33 [0.32, 0.35] (50/50)		0.2 [0.19, 0.21] (50/50)	-0.1 [-0.11, -0.09] (48/50)	-1.03 [-1.15, -0.92] (35/35)	
Ischaemic Heart Disease	48 hours	0.19 [0.16, 0.22] (46/50)					
IL15 (Preop)	Preop		0.13 [0.02, 0.24] (5/23)				
IL15 (48 hours)	48 hours			-0.07 [-0.08, -0.06] (47/50)			
IL1a (48 hours)	48 hours	-0.08 [-0.1, -0.07] (45/50)					
IL1b (48 hours)	48 hours					0.24 [0.06, 0.42]	

© 2024. Welch C et al. Published online at http://www.aginganddisease.org/EN/10.14336/AD.2024.0167

						(10/35)	
IL6 (Preop)	Preop			0.13 [0.03, 0.22] (8/23)			
IL7 (Preop)	Preop				0.17 [0.1, 0.24] (9/23)		
Leptin (48 hours)	48 hours	0.34 [0.33, 0.36] (50/50)		0.35 [0.34, 0.36] (50/50)	-0.33 [-0.34, -0.32] (50/50)		
Length of stay	48 hours					0.13 [0.05, 0.21] (15/35)	
Metformin	Preop				0.42 [0.17, 0.67] (10/23)		
Myostatin (48 hours)	48 hours	0.07 [0.06, 0.08] (36/50)					
Phase Angle (48 hours)	48 hours	0.05 [0.04, 0.06] (36/50)				-0.19 [-0.27, -0.12] (27/35)	
Resistin (48 hours)	48 hours	0.05 [0.04, 0.05] (36/50)				-0.12 [-0.23, -0.01] (7/35)	
Sex	48 hours	0.07 [0.06, 0.08] (45/50)		0.23 [0.21, 0.25] (50/50)	-0.07 [-0.08, -0.06] (44/50)		
Statin	Preop		0.07 [0.02, 0.12] (6/23)				
	48 hours				-0.1 [-0.12, -0.08] (39/50)		
Steroids	48 hours					0.78 [0.55, 1.01] (23/35)	
TNFa (48 hours)	48 hours	0.02 [0.01, 0.03] (19/50)			-0.02 [-0.03, -0.01] (6/50)		
TNFa (Preop)	Preop	0.17 [0.1, 0.23] (8/23)		0.15 [0.11, 0.19] (12/23)	-0.34 [-0.47, -0.21] (16/23)		
White Cell Count (48 hours)	48 hours						

Supplementary Table 5 – Beta coefficients derived from LASSO and Elastic Net models for change in outcomes from baseline, without specific focus on participants with additional systemic biomarkers available. Square brackets denote confidence intervals for coefficients. Curved brackets denote the number of models that the association was encountered within, and the number of models that the association was tested within. The timing of the individual variables and outcomes tested are denoted in the first column and row respectively. Variables without timing specified in the first

© 2024. Welch C et al. Published online at http://www.aginganddisease.org/EN/10.14336/AD.2024.0167

column are constants. The separate timing (second) column refers to the timing of other variables that the associated was tested against. Non-significant associations are not shown.

	Timing	ΔBATT (7 days)	ΔBATT (13 weeks)	ΔSMMSergi (7 days)	ΔEchogenicity (7 days)
Age	Preop	0.18 [0.13, 0.24] (7/36)	0.19 [0.16, 0.22] (27/36)		
	7 days	0.11 [0.09, 0.12] (68/79)			
Anxiety/ Depression	48 hours			-0.23 [-0.28, -0.18] (14/79)	
BATT (48 hours)	48 hours	-0.11 [-0.15, -0.07] (24/79)			
BATT (7 days)	7 days	0.4 [0.38, 0.42] (76/79)			
Cancer	Preop	0.17 [0.04, 0.29] (5/36)			-0.61 [-1.02, -0.21] (5/36)
	48 hours			0.19 [0.14, 0.24] (13/79)	
	7 days	0.14 [0.12, 0.16] (64/79)			
COPD	7 days	0.23 [0.21, 0.25] (71/79)			
Creatinine (7 days)	7 days			0.41 [0.37, 0.44] (11/79)	
Creatinine (Preop)	Preop				-0.14 [-0.23, -0.05] (6/36)
CRP (48 hours)	48 hours			0.13 [0.11, 0.15] (16/79)	
Delirium	Preop	-0.33 [-0.55, -0.11] (7/36)	-0.84 [-0.93, -0.75] (25/36)		
	7 days	-0.47 [-0.5, -0.44] (72/79)			
Diabetes Mellitus	48 hours			0.48 [0.38, 0.57] (14/79)	
Echo (48 hours)	48 hours			-0.16 [-0.2, -0.12] (21/79)	
eGFR (Preop)	Preop	-0.11 [-0.16, -0.05] (7/36)			0.24 [0.1, 0.38] (8/36)
eGFR (7 days)	7 days	-0.14 [-0.16, -0.13] (68/79)			
Hb (Preop)	Preop	-0.14 [-0.22, -0.06] (8/36)	-0.06 [-0.08, -0.04] (22/36)		

© 2024. Welch C et al. Published online at http://www.aginganddisease.org/EN/10.14336/AD.2024.0167

Hb (7days)	7 days	-0.04 [-0.05, -0.04] (55/79)		-0.03 [-0.05, 0] (5/79)	
Handgrip strength (48 hours)	48 hours			-0.06 [-0.08, -0.03] (13/79)	
Handgrip strength (7 days)	7 days			-0.05 [-0.08, -0.02] (7/79)	
Ischaemic Heart Disease	Preop		0.2 [0.14, 0.25] (25/36)		
	48 hours			-0.38 [-0.47, -0.29] (17/79)	
Length of stay	48 hours			-0.15 [-0.18, -0.12] (16/79)	
Metformin	48 hours			-0.54 [-0.67, -0.41] (15/79)	
Neutrophils (7 days)	7 days	0.12 [0.11, 0.14] (52/79)			
Phase Angle (48 hours)	48 hours			-0.2 [-0.24, -0.16] (23/79)	
PROMIS (7 days)	7 days	0.05 [0.04, 0.06] (54/79)			
SMMSergi (7 days)	7 days	-0.29 [-0.31, -0.27] (71/79)			
Non-smoker (vs current)	7 days	-0.15 [-0.17, -0.13] (65/79)			
	13 weeks	-0.15 [-0.22, -0.08] (4/79)			
Ex-smoker (vs current)	7 days	0.06 [0.04, 0.07] (47/79)			
Statin	Preop		0.48 [0.44, 0.51] (27/36)		
Steroids	Preop		-0.19 [-0.24, -0.15] (19/36)		
Stroke	7 days	0.29 [0.26, 0.32] (58/79)			
White Cell Count (7 days)	7 days	0.14 [0.12, 0.16] (50/79)			

Supplementary Table 6 – Beta coefficients derived from LASSO and Elastic Net models for change in outcomes from baseline, with specific focus on participants with additional systemic biomarkers available. Square brackets denote confidence intervals for coefficients. Curved brackets denote the number of models that the association was encountered within, and the number of models that the association was tested within. The timing of the individual variables and

© 2024. Welch C et al. Published online at http://www.aginganddisease.org/EN/10.14336/AD.2024.0167

SUPPLEMENTARY DATA

outcomes tested are denoted in the first column and row respectively. Variables without timing specified in the first column are constants. The separate timing (second) column refers to the timing of other variables that the associated was tested against. Non-significant associations are not shown.

	Timing	ΔBATT (7 days)	ΔBATT (13 weeks)	ΔSMMSergi (7 days)	ΔEchogenicity (7 days)
Anxiety/ Depression	Preop				-0.52 [-1.03, -0.01] (3/23)
Asthma	Preop	0.25 [0.07, 0.43] (3/23)			
COPD	Preop				0.35 [0.12, 0.58] (4/23)
Creatinine (48 hours)	48 hours			0.39 [0.31, 0.46] (6/50)	
Delirium	48 hours				0.13 [0.06, 0.2] (8/50)
Diabetes Mellitus	48 hours				-0.12 [-0.2, -0.03] (7/50)
eGFR (Preop)	Preop	-0.28 [-0.42, -0.14] (6/23)			
IL8 (48 hours)	48 hours				0.09 [0.07, 0.11] (11/50)
Leptin (48 hours)	48 hours				-0.11 [-0.17, -0.05] (10/50)
Leptin (Preop)	Preop			0.19 [0.04, 0.34] (5/23)	
Lymphocytes (48 hours)	48 hours				-0.12 [-0.16, -0.08] (11/50)
Metformin	Preop				0.21 [0.13, 0.28] (3/23)
Phase Angle (Preop)	Preop			-0.23 [-0.34, -0.13] (7/23)	
Sex (male)	Preop		-0.21 [-0.25, -0.16] (3/23)		
SMMSergi (48 hours)	48 hours				-0.18 [-0.24, -0.13] (9/50)
Steroids	48 hours				-0.25 [-0.34, -0.16] (9/50)
TNFa (48 hours)	48 hours			0.1 [0.07, 0.14] (7/50)	-0.15 [-0.19, -0.11] (12/50)
TNFa (Preop)	Preop			0.27 [0.12, 0.43] (12/23)	-0.27 [-0.4, -0.14] (5/23)
White Cell Count	48 hours				-0.09 [-0.11, -0.06]

© 2024. Welch C et al. Published online at http://www.aginganddisease.org/EN/10.14336/AD.2024.0167

(48 hours)					(6/50)

© 2024. Welch C et al. Published online at http://www.aginganddisease.org/EN/10.14336/AD.2024.0167
